# Abnormal functional lymphoid tolerance and enhanced myeloid exocytosis are characteristics of resting and stimulated PBMCs in cystic fibrosis patients

**DOI:** 10.3389/fimmu.2024.1360716

**Published:** 2024-02-26

**Authors:** Clémence Gaudin, Reem Ghinnagow, Flora Lemaire, Bérengère Villeret, Isabelle Sermet-Gaudelus, Jean-Michel Sallenave

**Affiliations:** ^1^ Laboratoire d’Excellence Inflamex, Institut National de la Santé et de la Recherche Medicale, Physiopathologie et Épidémiologie des Maladies Respiratoires, Université Paris-Cité, Paris, France; ^2^ INSERM, CNRS, Institut Necker Enfants Malades, Paris, France; ^3^ Université Paris-Cité, Paris, France; ^4^ ERN-LUNG CF Network, Frankfurt, Germany; ^5^ Centre de Ressources et de Compétence de la Mucoviscidose Pédiatrique, Hôpital Mignot, Paris, France

**Keywords:** cystic fibrosis, PBMCs, *Pseudomonas aeruginosa*, tolerance, proteases, lymphocyte, low-density neutrophils

## Abstract

**Introduction:**

Cystic Fibrosis (CF) is the commonest genetically inherited disease (1 in 4,500 newborns) and 70% of people with CF (pwCF) harbour the F508Del mutation, resulting in misfolding and incorrect addressing of the channel CFTR to the epithelial membrane and subsequent dysregulation of fluid homeostasis. Although studies have underscored the importance and over-activation of myeloid cells, and in particular neutrophils in the lungs of people with CF (pwCF), relatively less emphasis has been put on the potential immunological bias in CF blood cells, at homeostasis or following stimulation/infection.

**Methods:**

Here, we revisited, in an exhaustive fashion, in pwCF with mild disease (median age of 15, median % FEV1 predicted = 87), whether their PBMCs, unprimed or primed with a ‘non specific’ stimulus (PMA+ionomycin mix) and a ‘specific’ one (live *P.a* =PAO1 strain), were differentially activated, compared to healthy controls (HC) PBMCs.

**Results:**

1) we analysed the lymphocytic and myeloid populations present in CF and Control PBMCs (T cells, NKT, Tgd, ILCs) and their production of the signature cytokines IFN-g, IL-13, IL-17, IL-22. 2) By q-PCR, ELISA and Luminex analysis we showed that CF PBMCs have increased background cytokines and mediators production and a partial functional tolerance phenotype, when restimulated. 3) we showed that CF PBMCs low-density neutrophils release higher levels of granule components (S100A8/A9, lactoferrin, MMP-3, MMP-7, MMP-8, MMP-9, NE), demonstrating enhanced exocytosis of potentially harmful mediators.

**Discussion:**

In conclusion, we demonstrated that functional lymphoid tolerance and enhanced myeloid protease activity are key features of cystic fibrosis PBMCs.

## Introduction

Cystic Fibrosis (CF) is the commonest genetically inherited disease in the population (1 in 4,500 newborns) and 70% of people with CF (pwCF) harbour the F508Del mutation, which results in misfolding and incorrect addressing of the Cl- channel CFTR to the epithelial membrane (1–3) and subsequent dysregulation of fluid homeostasis on epithelial surfaces (4). In general, two (not mutually exclusive) main theories currently prevail to explain the inflammatory events associated with the disease: one suggests that there is an intrinsic defect in the mutated form of CFTR which activate inflammatory pathways and lung deterioration, independently of infectious stimuli (to name a few, refs 5–8). By contrast, the second states that the exacerbated inflammation found in CF is caused by microbial (*Pseudomonas aeruginosa (P.a)*, *Burkholderia cepacia*…) infections which thrive because of lack of CFTR (9–11) or following mucus accumulation in the lung mucosa (4, 12, 13). Apart from experimental evidence stemming from *in vitro* experiments where CFTR expression can be manipulated independently from infection (i.e. in transgenic or knock-out experiments in epithelial cell lines, eg refs 14, 15), this conundrum has been (and still is) extremely difficult to resolve in ‘real life’ situations in patients’ samples. Indeed, even in the absence of obvious on-going infections, past microbial ‘imprinting’ may interfere with experimental set-ups. In that context, on-going lung inflammation, through increased neutrophil elastase (NE) activity has been noted in young pwCF even in the absence of overt infection or disease (16–18). Interestingly, we showed that, relatedly, NE can disable CFTR activity *in vitro* and *in vivo*, in a murine animal model of *P.a* infection (19), thereby promoting an inflammatory vicious circle. This is to be put in the context of the well established neutrophilic phenotype in the lungs of pwCF and the findings that the Th2/Th17/Th22 pathways might be activated in that pathology (20–22). Indeed there has been growing interest in the role of immune cells in CF, especially in the lungs, where CFTR mutations have been associated with impaired pathogen clearance by myeloid cells (9), altered B-cell activation (23), and cytokine secretion by T-cells (24). However, the exact role that CFTR plays in modulating key immune cell functions remains unclear and identifying immune subsets in the CF lung, and key mechanisms of immunomodulation for therapeutic targeting (25) is of paramount importance.

Here, in that context, we wished to revisit, in a more exhaustive fashion, in a cohort of pwCF with mild disease (median age of 15, median % FEV1 predicted = 87, and who only 1/22 received CFTR modulators), whether their PBMCs, unprimed or primed with a ‘non specific’ stimulus (PMA+ionomycin mix) and a ‘specific’ one (live *P.a* =PAO1 strain), were already activated, when compared to healthy controls (HC) PBMCs.

Briefly, we:

1) comprehensively analysed by FACS the lymphocytic and myeloid populations present in CF and Control PBMCs (T cells, NKT, Tgd, NKs, ILCs, monocytes) and their production of the signature cytokines IFN-g, IL-13, IL-17, IL-22.
2) demonstrated by extensive q-PCR, ELISA and Luminex analysis that CF PBMCs have increased ‘background’ production of a wide variety of cytokines and mediators and a partial tolerance phenotype, when restimulated.
3) showed that CF PBMCs low-density neutrophils release higher levels of granule components (S100A8/A9, lactoferrin, MMP-3, MMP-7, MMP-8, MMP-9, NE), demonstrating increased exocytosis of potentially harmful mediators.

## Materials and methods

### Patients

Two groups of subjects were studied: 1) a ‘pwCF group’ (CF/median age = 15, n=22) with mild disease (median % FEV1 predicted = 87, where only one patient was under modulator (Orkambi) at the time of sampling); 2) a control group consisting of young healthy subjects (median age = 29, n= 9). Patients and healthy controls were matched for sex (50% and 55% male, respectively). The genetics, infectious and ventilatory status of all pwCF are listed in **Supplementary Table S1**. The Institutional review board for human studies of the APHP (Hôpital Necker, Assistance Publique des Hopitaux de Paris) approved the protocols and written consent was obtained from the subjects or their surrogates.

### Blood sampling and isolation of PBMCs

An average volume of 10 ml of blood samples from healthy subjects and pwCF was diluted ½ with PBS and deposited on 20 ml of Ficoll (Lymphocyte Seperation Medium, Sigma Aldrich). After 30 minutes of centrifugation at 800g without brake and at room temperature, the ring of PBMCs was recovered at the interface between the Ficoll and the serum. The cells recovered were washed in PBS, centrifuged for 10 minutes at 1500 g, then resuspended in 1 ml of PBS and counted on a Kova cell after a 1/10 dilution with trypan blue. Finally, 10 ^ 6 cells per 1 ml of 10% SVF-DMSO were placed in cryopreservation ampoules and stored at -80°C until use.

### Pseudomonas aeruginosa (PAO1 [ATC 15692]) culture

Two days before infection, bacteria (PAO1 strain) stored at -80° C were seeded on LB agar (lysogeny broth) and incubated overnight at 37° C. Bacteria were then precultured in 10 ml of liquid LB medium at 37° C, with overnight stirring. The pre-culture was then diluted (1/10^th^-1/5^th^) at 37° C with stirring for 1h30-2h in order to achieve exponential growth. PAO1 concentration was then determined with a spectrophotometer (densitometry at 600nm) and used to infect PBMCs (see below).

### PBMCs stimulation with PMA/ionomycin or infection with PAO1

PBMCs from control patients and pwCF stored at -80° C were thawed, washed in Dulbecco′s Modified Eagle′s Medium (DMEM) and centrifuged for 10 minutes at 1500 rpm. After counting with trypan blue on Kova cells, 2.10^6 cells were cultured (comprising approximately 1.2. 10^6 lymphocytes (T cells, B cells and NK cells) and 0.8.10^6 of myeloid CD11b+ cells) in DMEM free medium, in a 24-well cell culture plate (Corning ™ 3524) and stimulated with PMA/iono or infected with PAO1 at a multiplicity of infection of 1 (MOI). After 4 hours of stimulation/infection, PBMCs were recovered and centrifuged for 10 minutes at 1500g, cells were treated with RNA lysis buffer, or used for FACS analysis (see below). Simultaneously, supernatants (diluted ½ or ¼, in duplicates) were either analysed separately by ELISA (IL-1b, TNF-a, IL-8, IFN-g, IL-13, IL-17 DuoSet R&D Diagnostics) or pooled (within each HC or CF arm of the experiment) and analysed (40 mediators) using a customised Thermofisher Luminex Human Procartaplex Mix&Match (catalog numbers: PPX-35-MXXGTEK and PPX-14-MXNKTXF), according to the manufacturer’s instructions.

### RNA extraction and RT-qPCR

PBMC cells were lysed with lysis buffer containing 1% β-Mercaptoéthanol. RNA isolation was then performed with the PureLink RNA Mini Kit (12183018A, Ambion, Life Technologies, Asnières sur Seine, France), following the manufacturer’s instructions and as described previously (26).

qPCR primers were: HPRT= house-keeping gene:R: 5’ATCCAACACTTCGTGGGGTC3’; F: 5’ TTGCTTTCCTTGGTCAGGCA3’; TNF: R:5’ TGAGGTACAGGCCCTCTGAT 3’;F:5’CCCGAGTGACAAGCCTGTAG3’;IFNg:R:5’CTGGGATGCTCTTCGACCTC3’; F:5’AGTGATGGCTGAACTGTCGC3’; IL-13: R: 5’ GTCTCTGAACCCTTGGCTCC 3’; F:5’GTCTCTGAACCCTTGGCTCC3’; IL-22:R:5’CCAAGAGGGCCAAGAGAAGG3’;F: 5’ CAGTCACCAGTTGCTCGAGT 3’;IL-17:R5’GGATCTCTTGCTGGATGGGG3’; F: 5’ATCTCCACCGCAATGAGGAC3’**;**IL1b:R5’TCAACACGCAGGACAGGTAC3’;F:5’ GCTTGGTGATGTCTGGTCCA3’; IL-6: R:5’GGTCAGGGGTGGTTATTGCA

3’;F:5’CCAGAGCTGTGCAGATGAGT3’;IL8:R5’TCAGCCCTCTTCAAAAACTTCTC3’;F:5’CATACTCCAAACTTTCCACCCC3’;IL-10:R:5’ GCCACCCTGATGTCTCAGTTT; F:5’ACGGCGCTGTCATCGATTT;*P.a* opRL:R:5’ACCGGACGCTCTTTACCATA3’;F: CGAGTACAACATGGCTCTGG

Gene expression was expressed as: dCT = CT gene of interest – CT HPRT. Because dCT values are inversely correlated with gene expression levels, the Y axis of the PRISM panels are reversed, for a more intuitive visualisation of RNA expression (i.e. higher expression: ‘up’, lower expression: ‘down’). When appropriate, RNA expression from stimulated/infected- (with either PMA/iono or PAO1) HC or CF samples was expressed compared to their unstimulated control, using the formula: fold increase =2^-(ddCT).

### FACS analysis

FACS analysis of PBMCs (isolated post Ficoll gradient, see above) was performed for the quantification of the different cell types, using either membrane (CD45, CD3, CD4, CD8, CD11, CD14, CD15, CD16, CD66, CD19, NKp44, CD7, CD127, CD56, TCRgd, CD33), or intra-cellular markers (EOMES, IFNg, IL-13, IL-17, IL-22).

Briefly, 5.10^6^ to 10.10^6^ PBMCs were used for each condition, resuspended in 1 ml PBS, and the relevant mix of antibodies added (see **Supplementary Table S2**).

**Figure 1:**
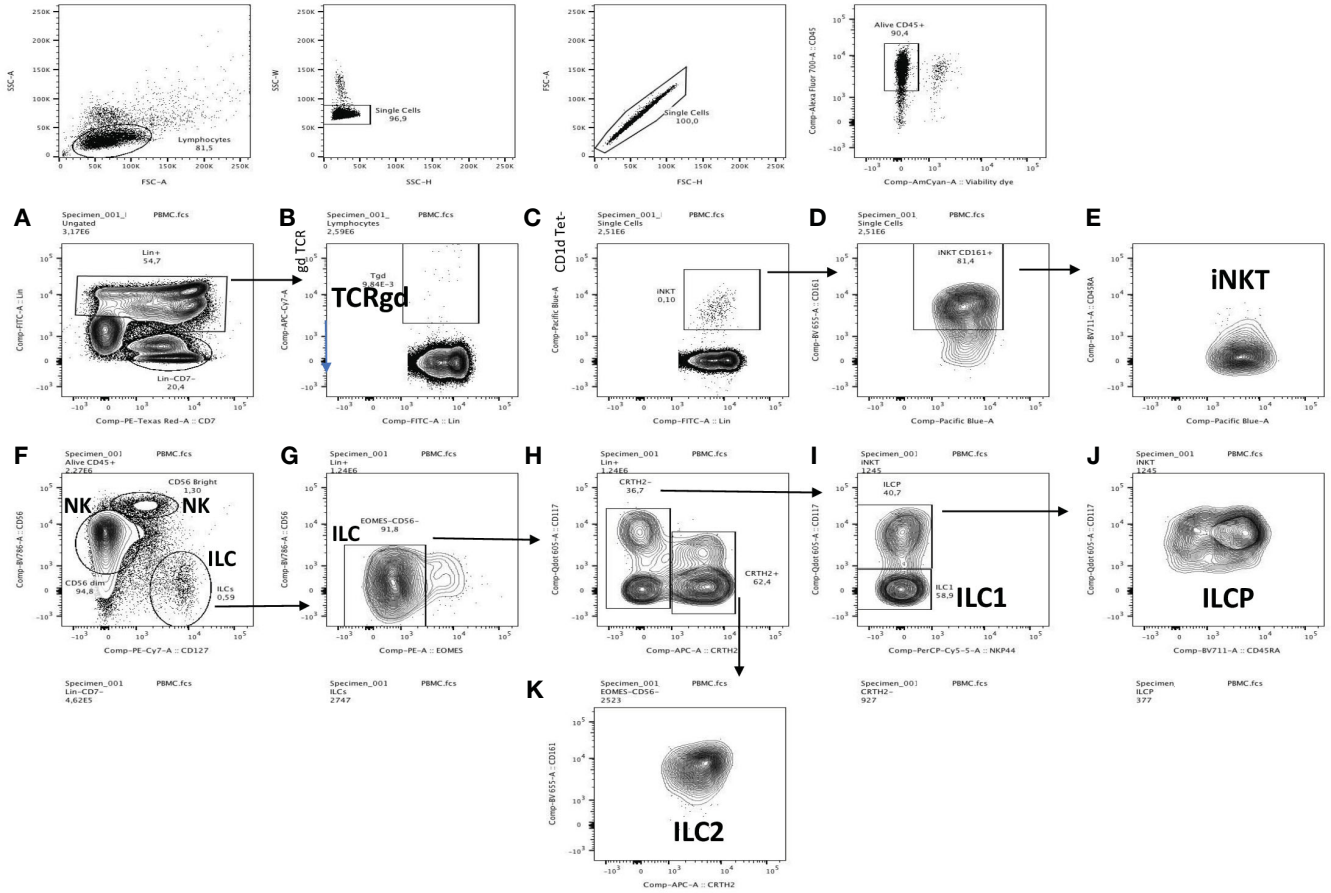
PBMC FACS gating strategy for the detection of total lymphocytes and monocytes (Lin +), TCRgd, NKT cells, NK dim, NK bright, ILC and ILCP cells. Singlets from a FSC/SSC lymphocytic gate were first selected, then gated for CD45+/viability, followed by cellular characterization using specific markers for lymphocytes and monocytes (Lin +, **A**), TCRgd **(B)**, NKT cells **(C–E)**, NK dim, NK bright **(F)**, ILC **(G–I, K)** and ILCP cells **(J)**. See Material and Methods for the detailed procedure.

**Figure 2:**
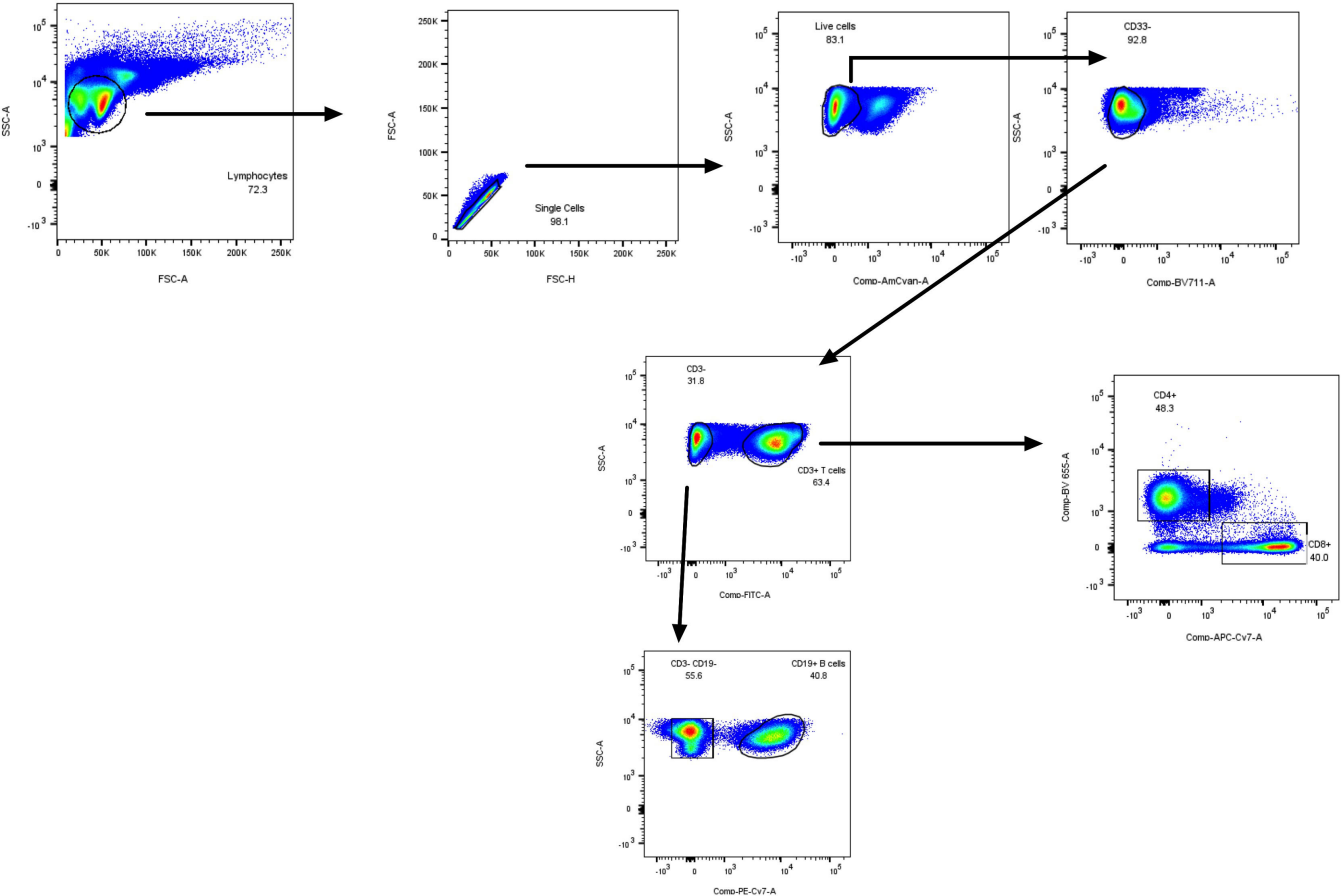
PBMC FACS gating strategy (lymphoid mix used in **Figure 3L**) for the specific detection of total T CD3+ lymphocytes, CD4+, CD8+ lymphocytes, and CD19+ B cells.

For membrane receptors analysis, 4 antibody mixes were used: the 1^st^ (**Figure 1**) was used to assess the PBMCs content of monocytes/CD14+, T (CD3), B lymphocytes (CD19) (all -FITC-labelled, named ‘Lin+’, panel A), as well as γδ T cells (panel B), NKT cells (panels C-E), NK CD56 Bright/dim cells (panel F) and innate lymphocytes (ILCs, panels F-K). The 2^nd^ was used to analyse separately T CD4+, T CD8+ and B cells (**Figure 2**). The 3^rd^ and 4^th^ mixes independently analysed monocytes (CD11b+CD14+) and neutrophils (CD15+CD66+) (**Supplementary Figure S1**).

For intra-cellular cytokines analysis, Golgi Plug was added prior to stimulation/infection of cells (BD Biosciences, 1/1000 dilution). Then, after 4hrs, one mix allowed for the assessment of IFN-γ, IL-13, IL-17 and IL-22 in Lin + cells (**Supplementary Figure S2A**), and in NK and ILC cells (panels B).

After incubation and/or permeabilization and washing, cells were fixed (Fix/Perm/eBioscience FoxP3 kit), centrifuged, washed (Perm Wash), and resuspended in PBS before analysis. For compensation purposes, mono-labelled tubes were used, using Ultra Comp eBeads (Invitrogen). Acquisition of events (around 20,000) were performed on an LSR-Fortessa and analysed with BD FACSDIVA™ and FlowJo (Treestar, OR) softwares.

### Proteolytic enzymatic activities in PBMC supernatants

PBMC supernatants enzymatic activities were analysed in 384 black wells plates in a final volume of 30 µL, using the procedure described (26). Briefly, neutrophil elastase (NE) activity was measured using the fluorogenic substrate Methoxysuccinyl-Ala-Ala-Pro-Val-7-amido-4-methylcoumarin, (Sigma), excitation and emission wavelength being 460 and 370 nm, respectively, while pan-metalloprotease activity was assessed with the substrate 5-FAM-Ser-Pro-Leu-Ala-Gln-Ala-ValArg-Ser-Ser-Ser-Arg-Lys(5-TAMRA)-NH2 (Enzo Life Sciences), excitation and emission wavelength being 485 and 535 nm, respectively. Fluorescence was read over a 3 h period, with a *Varioskan*™ *Flash Multimode Reader* (Thermo Scientific).

### Statistical analysis

Data were analysed with GraphPad PRISM software 9.3.1. Statistical analysis was performed with either t-tests, followed by non-parametric Mann-Whitney tests when two groups were compared, or by Kruskal Wallis tests, followed by *post-hoc* Dunn’s multicomparison tests when several groups were studied. A dendrogam/heatmaps analysis was also performed, encompassing all 40 Luminex analytes, using the ClustVis 2.0 program (https://biit.cs.ut.ee/clustvis/?s=mnEhQJZfoKkEurv). A heatmap was then obtained, using the same program.

## Results

### FACS analysis of PBMCs markers in healthy controls and CF patients

The cellular content of Ficoll-derived PBMCs from a population of young pwCF (see **Supplementary Table S1**) and of control young healthy patients (HC) was analysed by FACS. For both ethical and practical reasons, it was not possible to exactly age-match the two populations, with a median age of 15 (25% percentile =7.5) and 29 (25% percentile =24.0), for CF and HC subjects, respectively. To assess any potential age-related confounding factor, we correlated all measures of cell concentration (Lin+, TCRgd, iNKT, Lin-, CD56 Bright, CD56 Dim, total ILCs, ILC1, ILC2, ILCP) with the age of the HC subjects (**Supplementary Figure S3**) and that of pwCF (**Supplementary Figure S4**), and found no correlation, except for two variables (described in the paragraph below).

**Figure 3:**
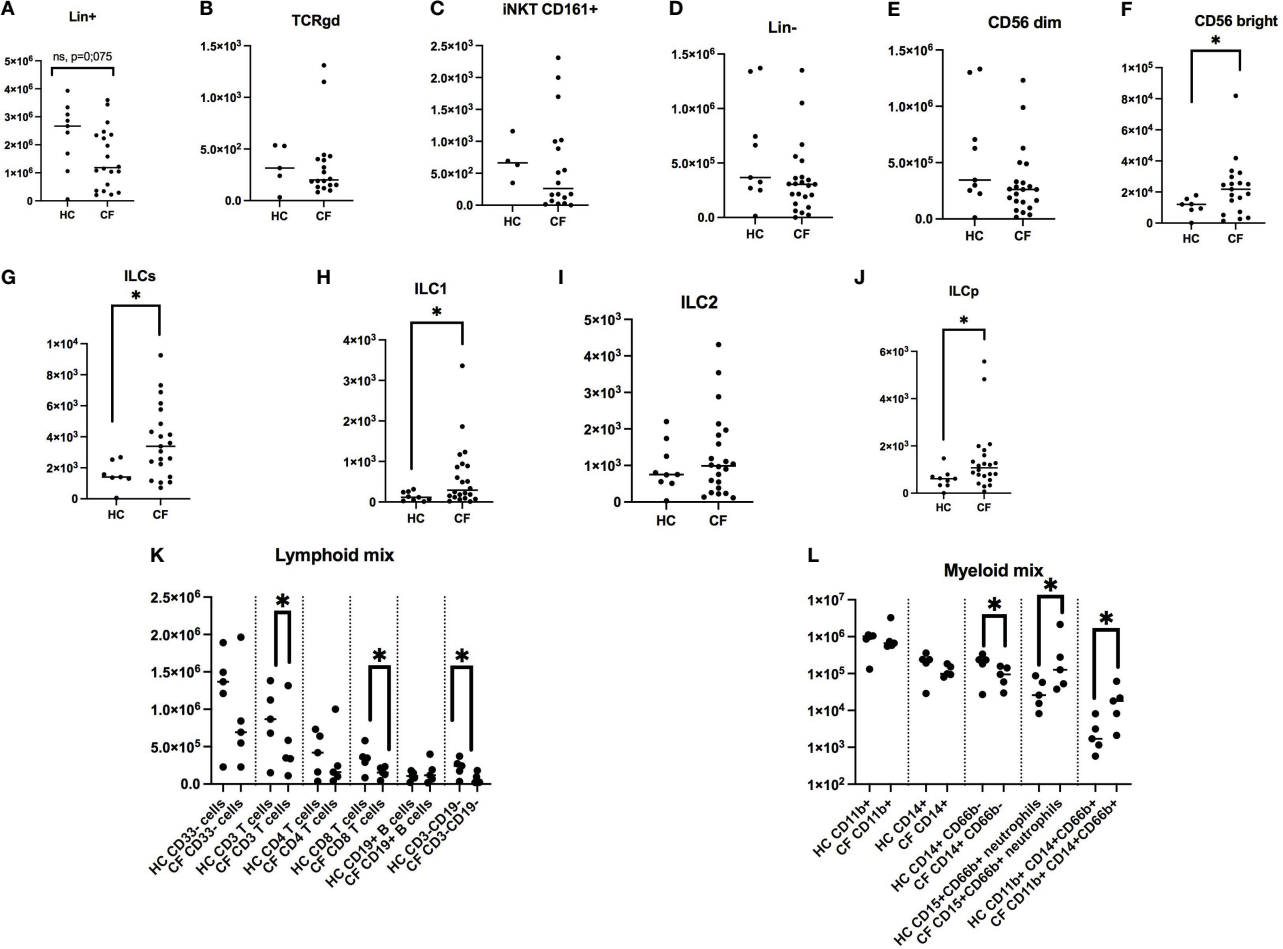
FACS analysis of PBMCs markers in healthy controls (HC) and people with CF. HC (n= 9) and CF (n= 22) PBMCs were analysed by FACS (see Materials and Methods). Numbers of Lin + cells (CD14+, CD3+, CD19+, all FITC-labelled) are shown in **(A)**. Within this population, the numbers of γδ T cells and NKT cells are depicted in **(B, C)**. Within Lin- cells **(D)**, the numbers of NK CD56 dim and CD56 bright are shown in **(E, F)**, respectively. Total ILCs and their sub-populations are shown in **(G–J)**. With independent antibody mixes, the of CD3+, CD4+, CD8+, CD19+ lymphocytes numbers are illustrated in **(K)**, whereas monocytes and neutrophils were analysed as shown in **(L)** (see **Supplementary Figure S1** for the gating strategy). Statistical analysis was performed with either t-tests, followed by non-parametric Mann-Whitney tests when two groups were compared, or by Kruskal Wallis tests, followed by *post-hoc* Dunn’s multi-comparison tests when several groups were studied. *: P<0.05; **: P<0.01; ***: P<0.001; ****: P<0.0001.

After appropriate gating of live CD45+ cells (see in **Figure 1** the FACS gating strategy), we analysed CD3+ CD4+ CD19 + CD14+ markers (all FITC-labelled, namely Lin+ cells) and showed, expectedly, that these cells (mostly TCR/BCR-bearing lymphocytes and monocytes) constituted the bulk of live CD45+ cells. Interestingly, there was a trend (p=0.07) for a reduction in Lin + cells and their % related to CD45+ cells in the CF group (**Figure 3A**, **Supplementary Figure S5A**). This down-regulation was not due to a reduction in either γδ T cells (3B, S5B) nor NKT cells (3C, S5C), as assessed in the same mix, but could be ascribed, as analysed with an independent mix (FACS strategy described in **Figure 2**) to ‘classical’ CD3+ T cells (**Figure 3L**, **Supplementary Figure S5L**) and, to a lesser extent to CD14+CD66- (most likely representing monocytes, **Figure 3M**, **Supplementary Figure S5M**). Interestingly, in CF (but not HC) PBMCs, there was a negative correlation between the % of Lin + and the age of the patients (**Supplementary Figure S4A**).

When total Lin- cells (i.e not TCR-bearing lymphocytes, nor monocytes) were considered (**Figure 3D**, **Supplementary Figure S5D**), CD56 dim ‘cytotoxic’ NK cells (**Figure 3E**) were, as expected, the remaining major cell population, independently of the phenotype, averaging between 10 and 15% of CD45+ cells (**Supplementary Figure S5E**), and there was no difference in NK concentration between HC and CF individuals. Interestingly, ‘inflammatory’ CD56 high NK cells (**Figure 3F**, **Supplementary Figure S5F**), which represent a small portion of total NKs, were increased in pwCF.

When total ILCs and their precursor ILCPs were analysed (**Figure 3G–J**, **Supplementary Figure S5G–K**), their proportion was relatively low (albeit higher than iNKT and Tgd cells, **Figures 1B, C**, **Supplementary Figure S5B, C**), and again pwCF had a higher proportion of these cells, compared to the Control group (**Figure 3G**, **Supplementary Figure S5G**). When ILCs were subdivided into ‘ILC1’, ‘ILC2’, ‘ILC3’, ‘ILCPs’, ILC3s were completely absent in both groups (not shown). When averaging ILCs, overall the % of ILC2s (**Supplementary Figure S5I**) surpassed that of ILC1s (roughly 4 fold, 4H), and there were obvious differences between the subject groups. Indeed, the % of HC ILC1s and ILC2s represented roughly 4% and 55% of all ILCs, respectively, whereas these proportions were 12.5% and 38% in pwCF, demonstrating a shift towards more ‘inflammatory’ ILCs in CF. Despite this overall trend, as for Lin+ cells (see above), there was a negative correlation between the % of ILC1s and the age of pwCF (**Supplementary Figure S4H**), and conversely, a positive correlation for HC ILC1s with age (**Supplementary Figure S3H**).

Remarkably, ILCPs, which are ‘poised’ precursor cells able to differentiate into ILCs at local mucosal sites, albeit at low concentration in peripheral blood (**Figure 3J**), represented 25% and 38% of HC and CF ILCs, respectively, suggesting an overall increased ILC- poiesis in CF blood (**Supplementary Figure S5K**).

Finally, we assessed the concentration of neutrophils, hereby named ‘low-density neutrophils’ (LDNs), given their presence in the upper band of Ficoll gradient (where PBMCs concentrate), and showed that their concentration was clearly increased in CF PBMCs, as assessed with the 2 neutrophil markers CD15 and CD66, **Figure 3L**).

Overall, this first part of our study demonstrated a clear phenotypic bias in CF PBMCs, with, quantitatively, a trend towards a reduction in the number and proportion of adaptive T cells and an increase in ‘pro-inflammatory cells’ (inflammatory CD56 Bright NK cells, ILCs, and LDNs).

### FACS analysis of HC and CF ‘lymphocytic’ cytokines (IFN-g, IL-13, IL-17, IL-22) following PMA/ionomycin stimulation of PBMCs

**Figure 4:**
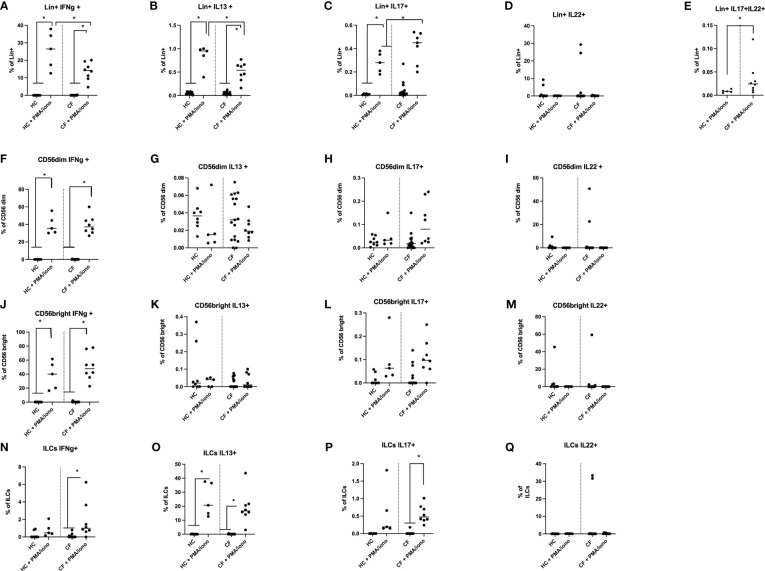
FACS analysis of HC and CF key ‘lymphocytic’ cytokines (IFN-g, IL-13, IL-17, IL-22) following PMA/ionomycin stimulation of PBMCs. HC (n=3-8) and CF (n=15-19) PBMCs were stimulated with a PMA/iono mix during 4hrs (see Materials and Methods) and analysed by FACS (see **Supplementary Figure S2** for the gating strategy). The intra-cellular production of IFN-γ, IL-13, IL-17 and IL-22 was assessed in Lin+ cells **(A–E)**, CD56 dim cells **(F–I)**, CD56 bright cells **(J–M)** and in total ILCs **(N–Q)**. Statistical analysis was performed with Kruskal Wallis tests, followed by *post-hoc* Dunn’s multi comparison tests. *: P<0.05; **: P<0.01; ***: P<0.001; ****: P<0.0001.

After assessing with appropriate markers the numbers and % of Lin+ and Lin- populations in PBMCs of HC and CF patients (see above), we set up to stimulate these PBMCs with a non specific stimulus (PMA/ionomycin), in a 4hrs protocol which did not induce cytotoxicity (not shown). An example of the FACS strategy (for Lin+, NK, Tgd and ILC cells) is depicted in **Supplementary Figure S2**. The intra-cellular levels of the cytokines IFN-g, IL-13, IL-17 and IL-22, signatures of ‘type1’, ‘type2’ and ‘type3’ responses, respectively, were then measured and quantitative results are shown in **Figure 4**.

Following PMA/ionomycin stimulation, irrespective of the ‘Healthy’ or ‘CF’ phenotype, there was in Lin+ cells (**Figures 4A–E**) a gradient of cytokine expression, with the type 1 cytokine IFN-g being the most prevalent cytokine, followed by IL-13, and IL-17. By contrast, IL-22 expression was more heterogeneous and was not PMA/ionomycin-responsive (**Figure 4D**).

In NK CD56 dim and CD56 Bright cells, the % of IFN-g+ cells was again prevalent (**Figure 4F**), post stimulation, compared to other cytokines (**Figures 4G, H, K, L**), with IL-22 expression being intermediate (**Figures 4I, M**).

Interestingly, ILC cells followed a different pattern, by producing mostly IL-13 in response to PMA/iono (about 20% of ILCs, **Figure 4O**), in keeping with the phenotypic characterization showing that ILC2s were prevalent among the ILCs (see **Figure 3I**). By comparison, the induction of IFN-g and IL-17 was less important (about 2 and 0.5% of ILCs, respectively, see **Figures 4N, P**), while there was no IL-22 induction in ILCs following PMA/iono stimulation (**Figure 4Q**).

When HC and CF PBMCs were compared side by side, there was, at homeostasis, no obvious difference between CF and HC PBMCs. However, following PMA/ionomycin stimulation, there was a reduction in the % of CF Lin+ PBMCs expressing IFN-g and IL-13 (**Figures 4A, B**), and conversely, an increase in CF in the % of Lin+ IL-17+ (**Figure 4C**) and Lin+ IL-17+IL-22+ cells (**Figure 4E**). For Lin- cells (CD56 and ILCs), no major differences were noted between Healthy and CF PBMCs upon PMA/iono stimulation.

### Transcriptional and translational analysis of key HC and CF PBMCs mediators post PMA/ionomycin stimulation

#### A) Cytokine mRNA production

**Figure 5:**
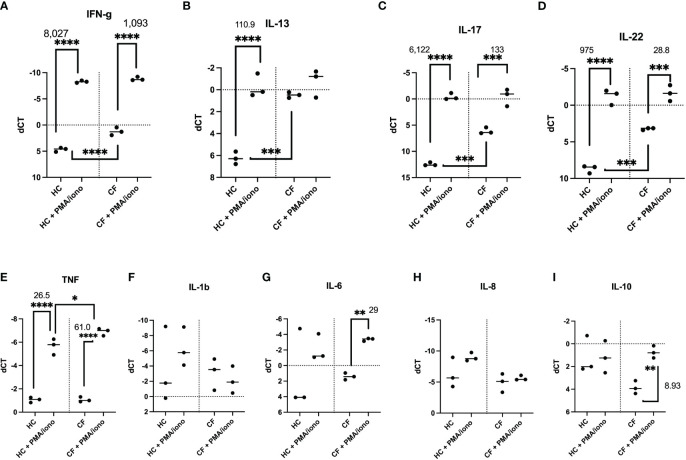
Transcriptional analysis of HC and CF PBMCs key cytokines post PMA/ionomycin stimulation. HC (n= 3) and CF (n= 3) PBMCs were either non-treated or stimulated with a PMA/iono mix during 4hrs, as in **Figure 4**. After cell lysis and RNA preparation (see Materials and Methods), IFN-γ, IL-13, IL-17, IL-22 **(A–D)**, TNF, IL-1b, IL-6, IL-8, IL-10 **(E–I)** RNA expression was measured by q-PCR. Gene expression is expressed as: dCT = CT gene of interest – CT house keeping gene. Because dCT values are inversely correlated with gene expression levels, the Y axis of the PRISM panels are reversed, for a more intuitive visualisation of RNA expression (i.e higher expression: ‘up’, lower expression: ‘down’). Statistical analysis was performed using either t-tests (when comparing +/- PMA/iono groups), or Kruskal Wallis tests, followed by *post-hoc* Dunn’s multi comparison tests, when all groups were compared, regardless of the treatment. *: P<0.05; **: P<0.01; ***: P<0.001; ****: P<0.0001. Numbers over the *symbols depict fold increase over HC- or CF- unstimulated cells.

Because the % of intra-cellular cytokine-expressing cells, as assessed by FACS, does not necessarily reflect protein expression and secretion in cell cultures, we then analysed the latter both at the transcriptional (q-PCR) and translational (Luminex and ELISA analysis of supernatants) levels, either basally or following a 4hrs stimulation with PMA/iono (**Figure 5**). Firstly, similarly to what was observed by FACS, and irrespective of the HC or CF phenotype, there was a gradient for the basal RNA expression of cytokines (IFN-g > IL-13 > IL-17 = IL-22, panels A-D). Secondly, CF PBMCs showed higher basal levels of IFN-g, IL-13, IL-17, IL-22 mRNA. Thirdly, PMA/iono up-regulated the mRNA of all these mediators, but the fold increase from the respective HC and CF controls (as assessed with the formula 2^(-ddCT) was invariably less pronounced in CF cells (medians of 1,093 v 8,027, 1.0 v 111, 133 v 6,122, 28.8 v 975 for IFN-g, IL-13, IL-17 and IL-22, respectively). This suggested that CF cells, upon stimulation, exhibited a relative ‘tolerance phenotype’ with respect to lymphocytic cytokines production.

Notably, differences between HC and CF were less obvious when some ‘key’ myeloid cytokines (IL-1b, TNF, IL-6, IL-8, IL-10) were considered (panels E-I), except for a low expression of IL-10 in CF cells, which was nevertheless inducible by the PMA/iono mix.

#### B) Cytokine protein secretion

**Figure 6:**
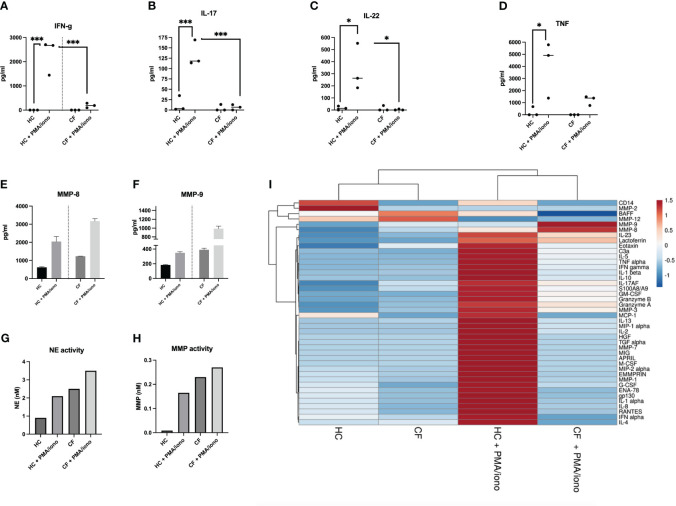
Protein analysis of HC and CF PBMCs post PMA/iono stimulation **(A–D)** HC (n=3) and CF (n= 3) PBMCs were stimulated with PMA/iono as in **Figure 4**. Cytokine levels for IFN-g, IL-17, IL-22, TNF were measured by ELISA in cell supernatants. Statistical analysis was performed using either t-tests (when comparing +/- PMA/iono groups), or Kruskal Wallis tests, followed by *post-hoc* Dunn’s multi comparison tests, when all groups were compared, regardless of the treatment. *: P<0.05; **: P<0.01; ***: P<0.001; ****: P<0.0001. Numbers over the *symbols depict fold increase over HC- or CF- unstimulated cells **(E, F)** HC (n=3) and CF (n= 3) PBMCs were stimulated as above. All supernatants from each category (either HC or CF) were harvested, pooled together and analysed by Luminex for MMP-8 and MMP-9 levels. **(G, H)** The same supernatants (as in **E, F**) were assessed for neutrophil elastase (NE) and metalloprotease (MMP) enzymatic activity, as described in M@M. **(I)**: The same supernatants (as in **E, F**) were analysed for a wide array of analytes (see **Supplementary Figures S6**-**S8**). A Cluster heatmap was then performed as described in M&M. Rows are centered, unit variance scaling being applied to rows. Both rows and columns are clustered using correlation distance and average linkage.

When PBMCs protein output of cytokines was considered, similar trends were observed, mirroring RNA data, with the induction in CF PBMCs of most cytokines being much less prominent, when compared to HC PBMCs (see representative mediators, as shown in **Figures 6A–D**/ELISA and a more extensive analysis (Luminex) encompassing ‘lymphoid cytokines (**Figure 6I**, **Supplementary Figure S6**), ‘myeloid cytokines’ (**Figure 6I**, **Supplementary Figure S7**), and ‘neutrophilic secretory granules markers’ (**Figure 6I**, **Supplementary Figure S8**).

Remarkably (and likely related to the increased numbers of LDNs in CF PBMCs), the only mediators present behaving differently, i.e demonstrating an increased response to PMA/iono stimulation in CF cells, were the metalloproteases MMP-8 and MMP-9 (present in neutrophil secondary and tertiary granules, respectively (**Figures 6E, F**). We also showed, in an independent assay, that the enzymatic activities of another important protease, the neutrophil elastase (NE, present in primary granules), were, like those of MMP-8 and MMP-9, increased in CF PBMCs, compared to HC cells, both at homeostasis, and following PMA/iono stimulation (**Figures 6G, H**).

### Transcriptional and translational analysis of key HC and CF PBMCs mediators post PAO1 infection

We then set up to assess, in an independent set of experiments, whether the behaviour of HC and CF PBMCs was similar when stimulated with a more ‘specific’ stimulus, i.e *P.a* live bacteria (PAO1 strain).

#### Cytokine mRNA production

**Figure 7:**
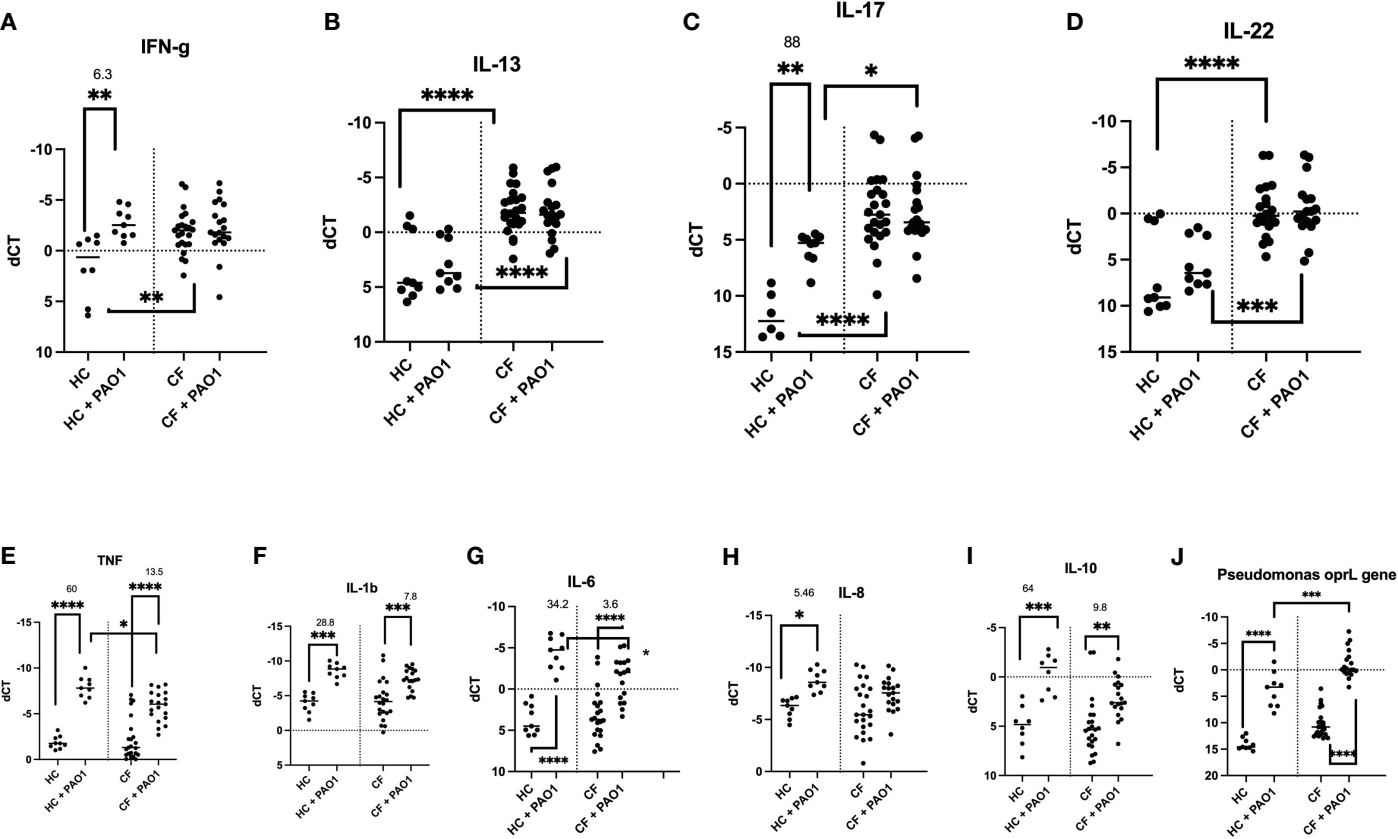
Transcriptional analysis of HC and CF PBMCs key cytokines, post live PAO1 infection. HC (n= 9) and CF (n= 22) PBMCs were either non-treated or infected with live *P.a* PAO1 strain (multiplicity of infection/MOI =1) during 4hrs. After cell lysis and RNA preparation (see Materials and Methods), IFN-γ, IL-13, IL-17, IL-22, TNF, IL-1b, IL-6, IL-8, IL-10 RNA expression was measured by q-PCR. **(A–I)** The levels of expression of the *P.a* gene opRL was used **(J)** as a read-out of PAO1 survival in PBMCs, as validated previously (27). Gene expression is represented as: dCT = CT gene of interest – CT house keeping gene (HPRT). Because dCT values are inversely correlated with gene expression levels, the Y axis of the PRISM panels are reversed, for a more intuitive visualisation of RNA expression (i.e higher expression: ‘up’, lower expression: ‘down’). Statistical analysis was performed as described in **Figure 5** legend. *: P<0.05; **: P<0.01; ***: P<0.001; ****: P<0.0001. Numbers over the *symbols depict fold increase over HC- or CF- unstimulated cells.

Notably, PAO1 did not induce the ‘lymphoid signature’ cytokines mRNA (IL-17, IL-13, IL-22, and IFN-g) in CF cells (**Figures 7A–D**), and their fold increase was also more modest in HC cells (6.3 and 88 for IFN-g and IL-17, respectively), when compared with that induced by PMA/iono (see **Figure 5** above).

When ‘pro-inflammatory’ mediators (TNF, IL-1b, IL-6, IL-8) were considered, PAO1 induced TNF, IL-1b, IL-6 mRNA in both HC and CF cells (**Figures 7E–G**), whereas IL-8 mRNA was poorly induced in CF PBMCs, compared to HC cells (**Figure 7H**). Interestingly, PAO1 induced the inhibitory cytokine IL-10 in both HC and CF PBMCs, but its fold-increase was reduced in CF cells (9.8 v 64, **Figure 7I**). Of note, CF PBMCs also exhibited an increased oprL PAO1expression (an index of PAO1 intracellular infection), potentially demonstrating a deficiency in PAO1 clearance in CF PBMCs (**Figure 7J**).

#### Cytokine protein secretion

**Figure 8:**
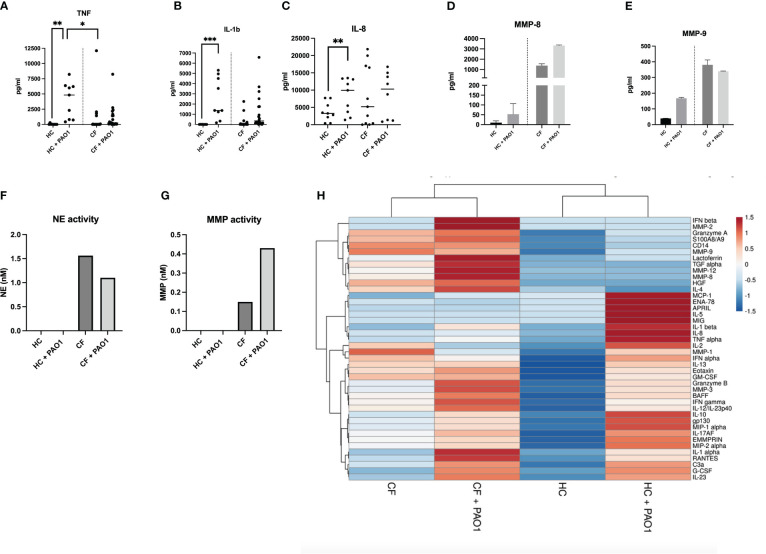
Protein analysis of HC and CF PBMCs post live PAO1 infection. **(A–C)** HC (n=3) and CF (n= 3) PBMCs were infected with live *P.a* PAO1 strain as in **Figure 7**. Cytokine levels for TNF, IL-1b, IL-8 were measured by ELISA in cell supernatants. Statistical analysis was performed as described in **Figure 5** legend**. (D–E)** HC (n=9) and CF (n= 22 PBMCs) were infected as above. All supernatants from each category (either HC or CF) were harvested, pooled together and analysed by Luminex for MMP-8 and MMP-9 levels. **(F, G)** The same supernatants (as in D-E) were assessed for neutrophil elastase (NE) and metalloprotease (MMP) enzymatic activity, as described in M@M. **(H)** The same supernatants (as in **D, E**) were analysed for a wide array of analytes (see **Supplementary Figures S9**-**S11**). A Cluster heatmap was then performed as described in M&M. Rows are centered, unit variance scaling being applied to rows. Both rows and columns are clustered using correlation distance and average linkage.

When PBMCs protein secretion was measured by Luminex and ELISA, the same trends were observed in CF cells. The levels of representative mediators are shown in **Figures 8A–E** (ELISA analysis) and also depicted are the results from a more extensive Luminex analysis.

The data show, as above, increased levels at homeostasis of many ‘lymphoid’ (**Figure 8H**, **Supplementary Figure S9**), ‘myeloid’ (**Figure 8H**, **Supplementary Figure S10**) cytokines and ‘neutrophilic secretory granules markers’ (**Figure 8H**, **Supplementary Figure S11**), and a reduced response towards PAO1 in CF cells (**Figure 8H**, **Supplementary Figures S9**-**S11**).

Notably, although present, this CF tolerance phenotype was globally less marked when compared to that observed with the PMA/iono stimulation, when comparing the ‘PAO1’and ‘PMA/iono’ heat maps (**Figures 8** and **6**, respectively).

Interestingly (and echoing what was observed with the less specific PMA/iono stimulation), there was, post PAO1, an increased production of neutrophilic granules components (S100A8/A9, lactoferrin, MMP-3, MMP-7, MMP-8, MMP-9, NE) in CF cells (**Figures 8D, E**, **Supplementary Figure S11**, and a similarly enhanced enzymatic activity of NE and metalloproteases (**Figures 8F, G**), correlated with the (maintained) high induction of G-CSF (a key regulator of neutrophil production) in CF PBMCs (**Supplementary Figure S10K**).

## Discussion

Although the potential immune bias towards Th1/Th2/Th17 responses in CF lungs has been studied extensively, in both murine models and in humans (to name a few, refs 20–22, 27–30), this has been much less investigated in peripheral blood cells.

Indeed, before the advent of the Th1/Th2/Th17 classification, some earlier studies indicated that blood T helper cell function might be deficient in CF (31–33), but relatively few studies have since then tackled the potential immune bias of CF blood cells, at homeostasis or following stimulation/infection.

Relatedly, it has been shown that normal lymphocytes do express CFTR (24), but whether a mutant CFTR has functional consequences on human T cell activity is still an open question (28, 34).

Mulcahy et al. (35) suggested that high peripheral blood Th17% was associated with poor lung function in CF and Kushwah et al. (36) proposed that naïve CF cells have an intrinsic ability to differentiate towards a Th17 phenotype. Few studies, however, have used *P.a* as a recall antigen in lymphocytic proliferation assays (37, 38) and live *P.a* was seldomly used (38).

In the current study, we compared, in what we believe is the most thorough analysis of CF PBMCs in a single study, T cells (Tαβ CD4/CD8, Tγδ, NKT), B cells, NK cells (CD56bright/CD56dim), ILCs (39), monocytes and low-density neutrophils with their HC counterparts. As expected, Lin+ T cells/monocytes were the most prevalent cells in both HC and CF PBMCs, followed by NK cells, and in roughly the same proportions Tγδ, NKT and ILC cells. We showed that there was a trend towards a reduction in the number and proportion of Lin+ cells in CF cells, and that conversely, those of ‘innate inflammatory cells’ CD56 bright NK cells, ILCs and LDNs were increased (**Figure 3**, **Supplementary Figure S5**).

Phenotypically, we showed by FACS analysis that the main difference in intra-cellular cytokine production between PMA/iono-stimulated HC and CF PBMCs resided in the Lin+ T cell population, with overall decreased cytokine inducibility in CF cells (**Figure 4**), except for IL-17+IL-22+ cells, even though they accounted for a very low percentage of total Lin+ cells (0.01-0.04%), overall mirroring the described Th17 phenotype in pwCF.

This potential tolerance phenotype was confirmed at the transcriptional and protein levels (**Figures 5**, **6**, **Supplementary Figure S6**-**S8**), when all cells (and not only Lin+ cells) were analysed, showing increased background levels of cytokines in CF PBMCs, combined to a reduced inducibility by PMA/iono, with the notable exception of IL-10, at the RNA level (**Figure 5**).

We believe that one of the originalities of our study also lies in the study of live PAO1 as a stimulus in addition to PMA/iono. Indeed, few studies have used the latter, and most have used dead *P.a* extracts, or isolated virulence factors to assess CF PBMC responses (37, 40–43).

Importantly, when characterizing immune tolerance following *P.a* infection, caution is warranted since it is well established, as shown by us (26, 27, 44) and others, that *P.a* can release many virulence factors such as proteases (eg LasB), able to degrade some immune mediators, but not others (45–49).

Therefore, the decreased secretion of a given cytokine, following *P.a* infection, should probably only be characterized as a consequence of tolerance if two conditions are fulfilled: firstly with an increase in basal (i.e in the absence of *P.a* infection) cytokine RNA and protein levels in CF, compared to HC PBMCs, and secondly a reduced PAO1-driven induction of cytokine RNA, with or without a similar decrease at the protein level.

Importantly, the fact that this functional tolerance is not a ‘pan-immune’ phenomenon is best exemplified here by the finding that CF PBMCs low-density neutrophils release higher levels of granule components (S100A8/A9, lactoferrin, MMP-3, MMP-7, MMP-8, MMP-9, NE), following both PMA/iono and PAO1 stimulation (**Figure 8**, **Supplementary Figures S8**, **S11**).

This, correlated with the higher levels of G-CSF produced at homeostasis, demonstrates enhanced exocytosis in CF PBMCs and potentially, increased deleterious pro-inflammatory activity, which may ultimately be transferred to CF lung tissue (50). This overall increased granule exocytosis and concomitant inhibition of cytokine expression in CF PBMCs post stimulation is interesting and will require further studies to decipher the mechanisms at play. Indeed, because we used here total PBMCs and did not further purify their different components, it is in principle difficult to distinguish whether CF mononuclear cells and neutrophils are ‘per se’ intrinsically different eg in their receptor collection (explaining their differential responses to PMA/iono or *P.a*), or whether soluble factors might act ‘in trans’ to affect their function. Nevertheless, it is noteworthy that overall, CF PBMCs respond more to *P.a* than to PMA/iono (clearly shown in the heatmaps in **Figures 8** and **6**, respectively), hinting towards the idea that the CF lymphocytic intra-cellular ‘adaptive immune’ machinery (the target of PMA/iono stimuli) might be deficient. Equally, as pointed above, the fact that neutrophils exocytosis markers were among the rare mediators that were significantly induced by both PMA/iono and *P.a*, again suggests that CF neutrophils may also be intrinsically different.

Although an increasing number of studies describe the presence of low-density neutrophils in PBMCs, their role in diseases remains controversial. Indeed, in a different context, their immunosuppressive activity has been put forward as important for the maintenance of tumorigenicity (51), but their potential importance in infectious diseases is comparatively a relatively new and more emerging concept (52–57).

Interestingly, recent data demonstrate that myeloid cell activity might be modulated by CFTR modulators (25, 58). However, whether the abnormalities of CF PBMCs observed here can partially or totally be corrected with modulators/correctors was not possible to assess in our study since 21/22 of PBMCs were obtained from ‘modulator-free’ patients. Potentially, when and if ethical considerations make it possible, detailed phenotypic studies of PBMCs before and after treatment may be useful to determine whether modified cellular phenotypes are indicative of drug efficacy.

Regardless, we believe that the dichotomy present in CF PBMCs, i.e functional tolerance of the lymphoid compartment, and enhanced exocytosis activity in low-density neutrophils following stimulation/infection with *P.a* may provide a rationale for therapeutic avenues for patients ineligible to modulator’s therapy, through the boosting of adaptive immunity and the provision of an anti-inflammatory regimen.

## Data availability statement

The raw data supporting the conclusions of this article will be made available by the authors, without undue reservation.

## Ethics statement

The studies involving humans were approved by Institut Necker Enfants Malades-APHP. The studies were conducted in accordance with the local legislation and institutional requirements. Written informed consent for participation in this study was provided by the participants’ legal guardians/next of kin.

## Author contributions

CG: Data curation, Methodology, Software, Writing – review & editing. RG: Data curation, Methodology, Software, Writing – review & editing, Investigation. FL: Data curation, Investigation, Methodology, Software, Writing – review & editing. BV: Data curation, Investigation, Methodology, Software, Writing – review & editing. IS-G: Writing – review & editing. J-MS: Writing – review & editing, Conceptualization, Data curation, Formal analysis, Funding acquisition, Investigation, Methodology, Project administration, Software, Supervision, Validation, Writing – original draft.

## Supplementary material

Supplementary Table S1 CF patients: Demographic, genetic, infectious and clinical status. NA: ‘not available’.

Supplementary Table S2 FACS antibodies used in the analysis of PBMCs from HC and CF subjects.

Supplementary Figure S1 PBMC gating strategy (myeloid mix used in **Figure 3M**) for the specific detection of CD14+ monocytes and CD14-CD15+CD66+ neutrophils.

Supplementary Figure S2 PBMC gating strategy (for antibody mixes used in **Figure 4**) for the intra-cellular detection of IFN-g, IL-13, IL-17, IL-22 in Lin+ cells (monocytes and lymphocytes), CD56 bright, CD56 dim, ILC cells.

Supplementary Figure S3 Correlation between the age of HC subjects and the % of cells as detected by FACS (**Figures 1**, **3**).

Supplementary Figure S4 Correlation between the age of CF patients and the % of cells as detected by FACS (**Figures 1**, **3**).

Supplementary Figure S5 FACS analysis of PBMCs markers in healthy controls (HC) and people with CF .The same analysis as in **Figure 3** was performed, except that numbers are expressed as % relative to CD45+ cells.

Supplementary Figure S6 Lymphoid cytokine production by PBMCs post PMA/ionomycin stimulation. HC (n= 3) and CF (n= 3) PBMCs were stimulated with PMA/iono during 4hrs as described in **Figure 6** and in Materials and Methods. Supernatants were recovered and ‘lymphoid cytokines’ levels were analysed by Luminex

Supplementary Figure S7 Myeloid cytokine production by PBMCs post PMA/ionomycin stimulation. The same supernatants generated as explained in **Supplementary Figure S6** were analysed by Luminex for assessing ‘myeloid cytokines’ levels.

Supplementary Figure S8 Neutrophilic secretory granules markers production by PBMCs post PMA/ionomycin stimulation. The same supernatants generated as explained in **Supplementary Figure S6** were analysed by Luminex for assessing ‘neutrophilic secretory granules’ levels.

Supplementary Figure S9 Lymphoid cytokine production by PBMCs post live PAO1 infection. HC (n= 9) and CF (n= 22) PBMCs were infected with live PAO1 during 4hrs as described in **Figure 7** and in Materials and Methods. Supernatants were recovered and ‘lymphoid cytokines’ levels were analysed by Luminex.

Supplementary Figure S10 Myeloid cytokine production by PBMCs post live PAO1 infection. The same supernatants generated as explained in **Supplementary Figure S8** were analysed by Luminex for assessing ‘myeloid cytokines’ levels.

Supplementary Figure S11 Neutrophilic secretory granules markers production by PBMCs post live PAO1 infection. The same supernatants generated as explained in **Supplementary Figure S9** were analysed by Luminex for assessing ‘neutrophilic secretory granules’ levels.
