# Supplementary material for: Abnormal functional lymphoid tolerance and enhanced myeloid exocytosis are characteristics of resting and stimulated PBMCs in cystic fibrosis patients
Source: Front Immunol. 2024 Feb 26;15:1360716. doi: 10.3389/fimmu.2024.1360716 (PMC10925672; doi:10.3389/fimmu.2024.1360716)
Supplement: Supplementary Table S1 — CF patients: Demographic, genetic, infectious and clinical status. NA: ‘not available’. [file Table_1.docx]

| **CF** | age | gender | mutation | Pathogens at date of sampling | Modulators at time of sampling | FEV1 predicted (%) |
| --- | --- | --- | --- | --- | --- | --- |
| 1 | 18 | male | Phe508del /394delTT | *S.aureus/P.aeruginosa*/*mycobacterium.avium*/ | NO | 59 |
| 2 | 17 | female | W1282X/17a17b-18del | *S.aureus, P.a* | NO | 120 |
| 3 | 8 | female | W1098X/G542X | *S.aureus MS* | NO | NA |
| 4 | 4 | female | E1104X/ΔF508 | *P.a, Aspergillus* | NO | NA |
| 5 | 8 | male | ΔF508del/exon 6a6b | *Maltophilus* | NO | 89 |
| 6 | 2.5 | male | G542X/542X | *S.aureus* | NO | NA |
| 7 | 15 | male | ΔF508del/G542X | *S.aureus* | NO | NA |
| 8 | 15 | male | ∆ F508/G542X | *H.Influenzae + S.aureus MS* | NO | 73 |
| 9 | 6 | female | ΔF508+/+ | *S.aureus* | NO | NA |
| 10 | 13 | female | ΔF508+/+ | *S.aureus* | NO | 68 |
| 11 | 7 | female | ∆ F508/2789 + 5 G > A | no | NO | 118 |
| 12 | 12 | female | ΔF508+/+ | *S.aureus* | NO | 107 |
| 13 | 19 | male | ΔF508/1717-1G>A | *S.aureus* | NO | 78 |
| 14 | 18 | male | 711+1G>T/3120+1G>A | *S.aureus* | NO | 76 |
| 15 | 15 | male | ΔF508/G542X | *S.aureus* | NO | 87 |
| 16 | 19 | female | ΔF508/G542X | *S.aureus* | NO | 103 |
| 17 | 16 | female | ΔF508+/+ | *P.a* | NO | 60 |
| 18 | 16 | male | G551D/TG12T5 | *S.aureus* | NO | 102 |
| 19 | 15 | female | ΔF508+/+ | *S.aureus* | Orkambi | 85 |
| 20 | 11 | female | G1244E/R352Q | *S.aureus* | NO | 118 |
| 21 | 7.5 | male | ∆F508/dele2,3(21kb) | *S.aureus* | NO | 85.2 |
| 22 | 17 | male | ∆ F508/∆ F508 | *S.aureus* | NO | 129 |
|  |  |  |  |  |  | Median : 87 |

TABLE S1
