# Supplementary material for: Abnormal functional lymphoid tolerance and enhanced myeloid exocytosis are characteristics of resting and stimulated PBMCs in cystic fibrosis patients
Source: Front Immunol. 2024 Feb 26;15:1360716. doi: 10.3389/fimmu.2024.1360716 (PMC10925672; doi:10.3389/fimmu.2024.1360716)
Supplement: Supplementary Table S2 — FACS antibodies used in the analysis of PBMCs from HC and CF subjects. [file Table_2.docx]

| **Antibody and beads** | **Fluorochrome** | **Supplier** | **Batch** | **Microliter/well** |
| --- | --- | --- | --- | --- |
| Viability Dye | eFluor 506 | Invitrogen | 65-0866-14 | 1 |
| CD3 | FITC | Invitrogen | 4341630 | 1 |
| CD4 | FITC | Biolegend | B234953 | 1 |
| CD4 | BV650 | Biolegend | B366745 | 0.5 |
| CD8 | APC-Cy7 | Biolegend | B355317 | 0.5 |
| CD14 | FITC | Miltenyi Biotec | 5171109352 | 1 |
| CD11b | APC-Cy7 | Biolegend | B367906 | 0.5 |
| CD66b | PE-Cy7-A | Biolegend | B377326 | 0.5 |
| CD15 | BV-510-A | Biolegend | B360125 | 0.5 |
| CD16 | BV711-A | Biolegend | B362363 | 0.25 |
| CD19 | FITC | Miltenyi Biotec | 5171124418 | 1 |
| NKp44 | PerCP-Cy5.5 | Biolegend | B229049 | 2.5 |
| CD7 | PE-CF594 | BD Biosciences | 7038863 | 1 |
| CD127 | PE-Cy7 | Invitrogen | 4329889 | 1 |
| CD45 | AF700 | BD Biosciences | 7242993 | 1 |
| CD117 | BV605 | Biolegend | B239327 | 2.5 |
| CD56 | BV786 | Biolegend | B243205 | 1.5 |
| TCRgd | APC-Cy7 | Miltenyi Biotec | 5180228472 | 0.5 |
| iNKT | BV421 | Biolegend | B250074 | 1 |
| CD161 | BV650 | BD Biosciences | 7235832 | 2.5 |
| CRTh2 | AF647 | Biolegend | B220661 | 2.5 |
| CD45A | BV711 | BD Biosciences | 7269783 | 0.5 |
| CD33 | BV711A | Biolegend | B280391 | 0.5 |
| EOMES | PE | Invitrogen | 4327160 | 1.5 |
| IFNg | BV650 | Biolegend | B238336 | 2.5 |
| IL-13 | BV421 | BD Biosciences | 7194781 | 2 |
| IL-17A | BV711 | Biolegend | B241946 | 1.5 |
| IL-22 | eFluor 660 | Invitrogen | 1917787 | 2 |
| Ultra comp beads | - | Invitrogen | 01-2222-42 | 1 |
| Golgi Plug |  | BD Biosciences | 555029 | 1 |

**Table S2**
