# Supplementary figures and images for: Abnormal functional lymphoid tolerance and enhanced myeloid exocytosis are characteristics of resting and stimulated PBMCs in cystic fibrosis patients
Source: Front Immunol. 2024 Feb 26;15:1360716. doi: 10.3389/fimmu.2024.1360716 (PMC10925672; doi:10.3389/fimmu.2024.1360716)

## Slide 1
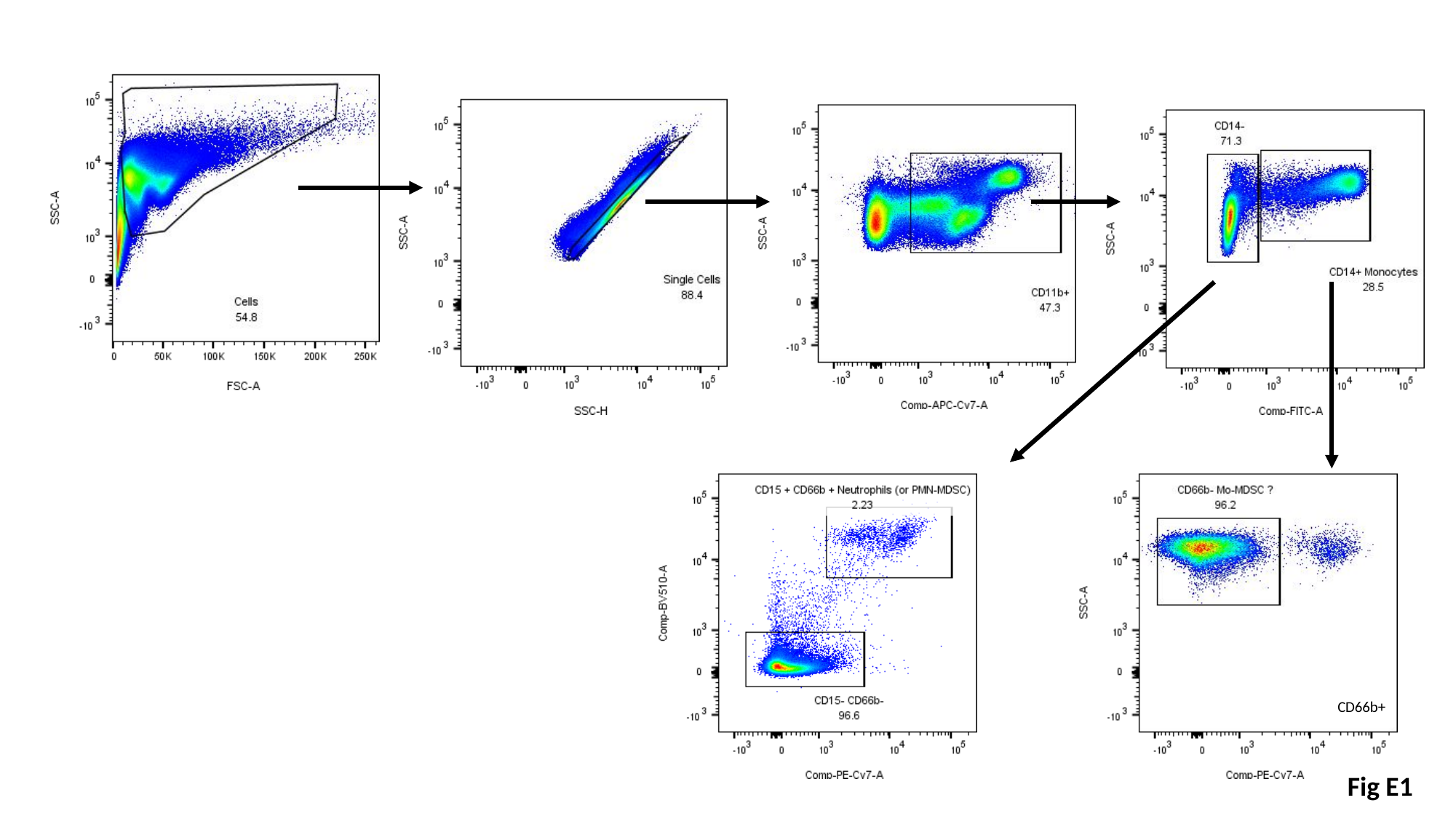

CD66b+
Fig E1

Supplement: Supplementary Figure S1 — PBMC gating strategy (myeloid mix used in Figure 3M ) for the specific detection of CD14+ monocytes and CD14-CD15+CD66+ neutrophils. [file Presentation_1.pptx]

## Slide 1
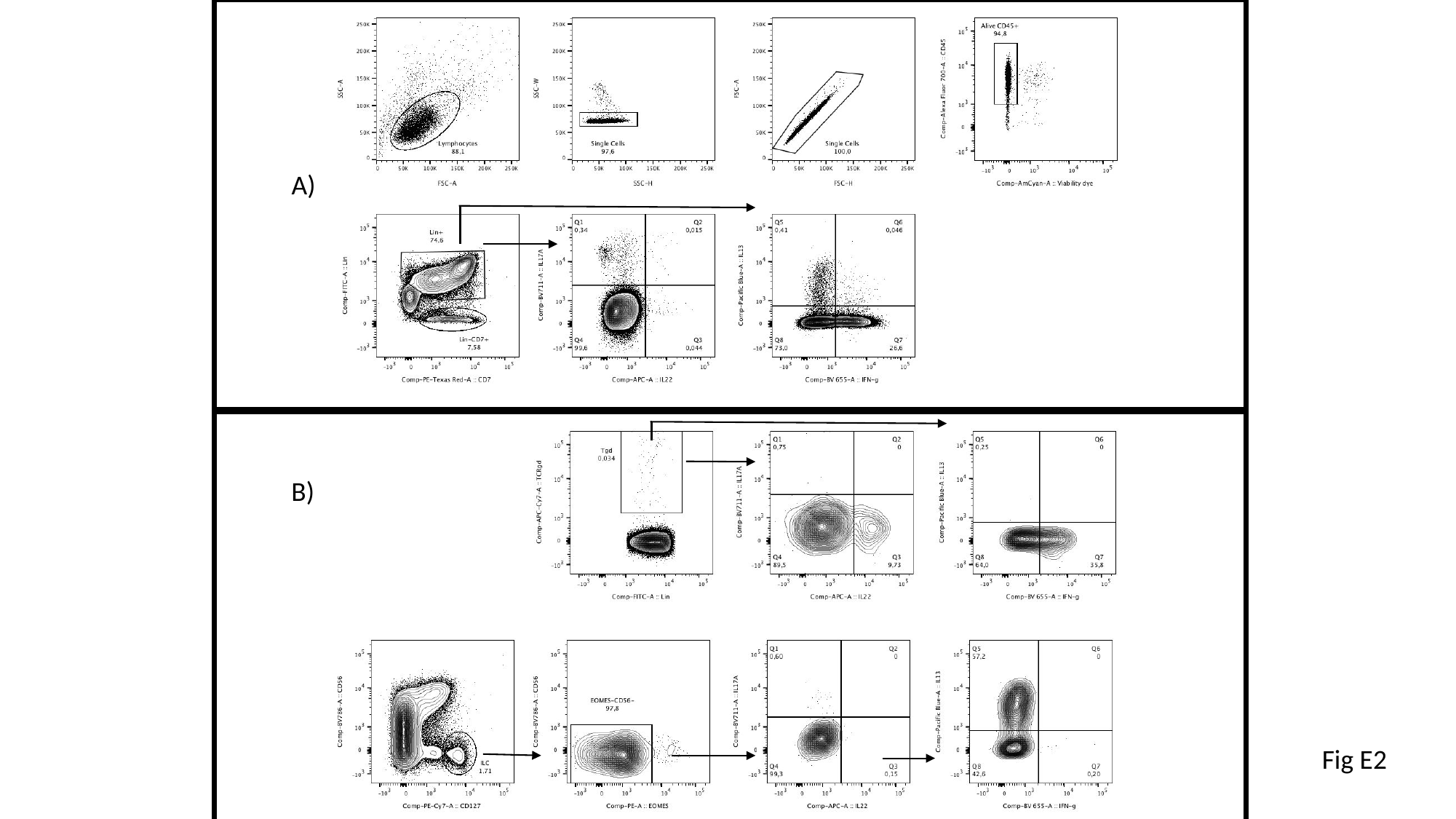

A)
B)
Fig E2

Supplement: Supplementary Figure S2 — PBMC gating strategy (for antibody mixes used in Figure 4 ) for the intra-cellular detection of IFN-g, IL-13, IL-17, IL-22 in Lin+ cells (monocytes and lymphocytes), CD56 bright, CD56 dim, ILC cells. [file Presentation_2.pptx]

## Slide 1
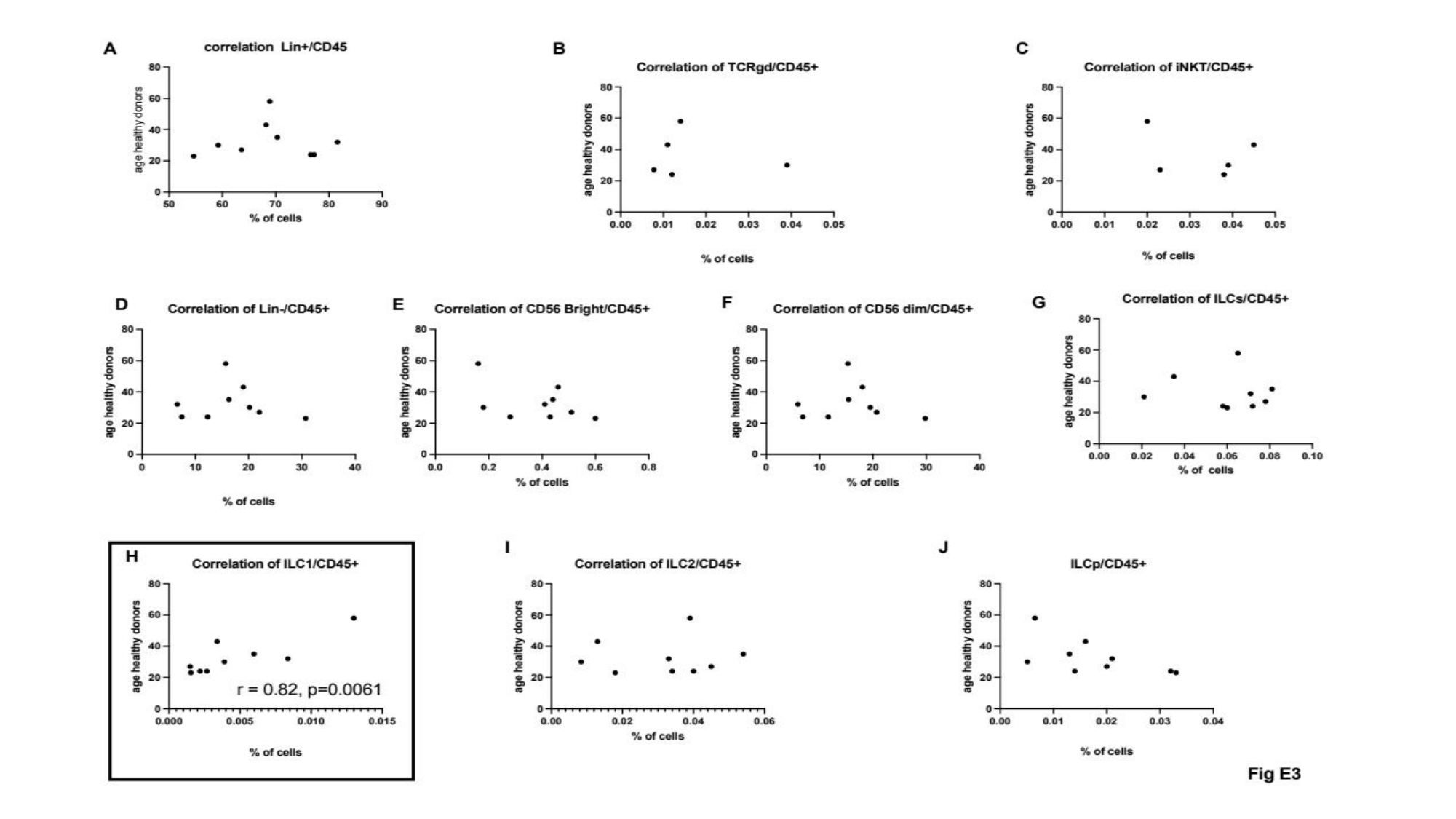

Supplement: Supplementary Figure S3 — Correlation between the age of HC subjects and the % of cells as detected by FACS ( Figures 1 , 3 ). [file Presentation_3.pptx]

## Slide 1
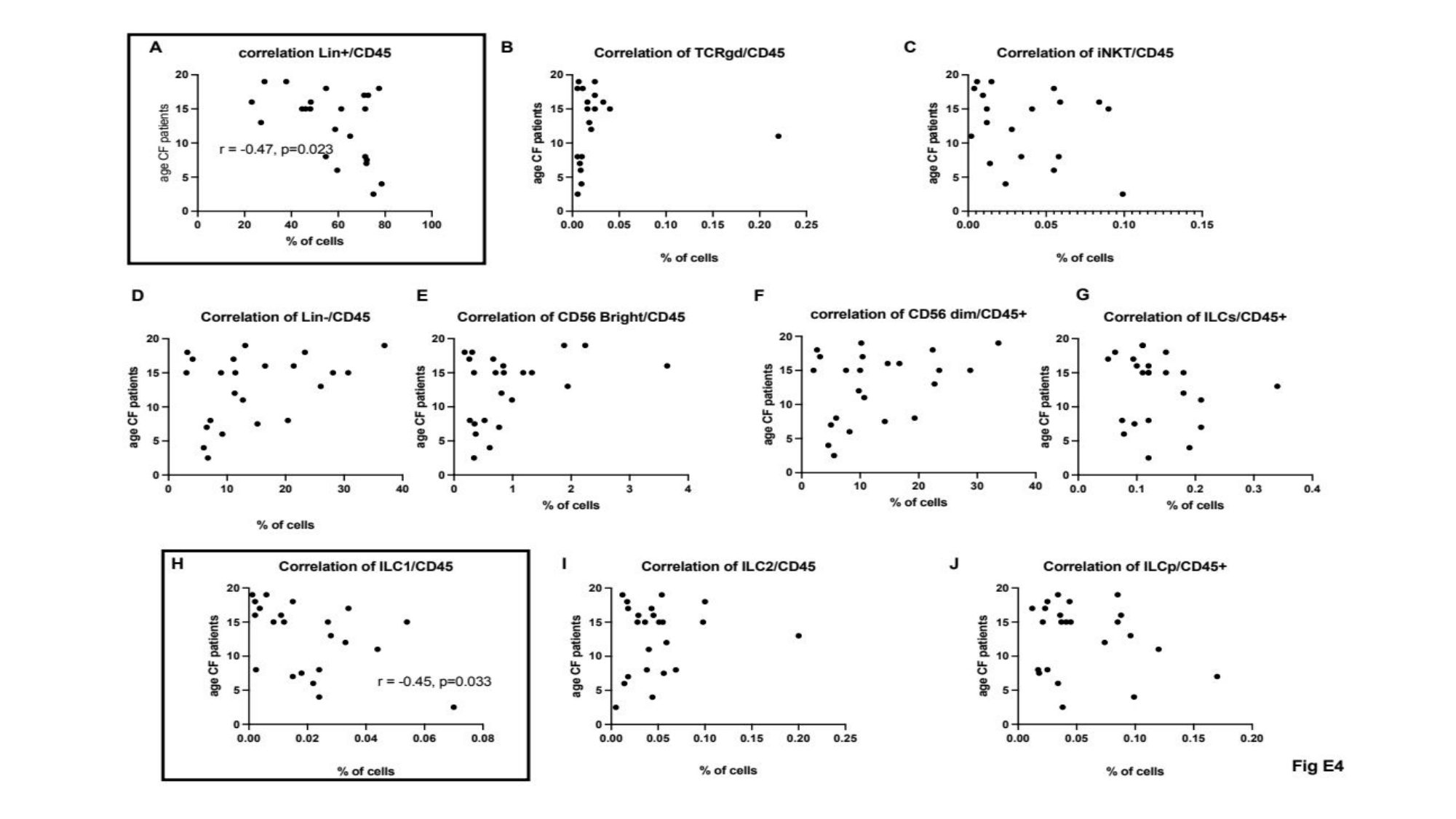

Supplement: Supplementary Figure S4 — Correlation between the age of CF patients and the % of cells as detected by FACS ( Figures 1 , 3 ). [file Presentation_4.pptx]

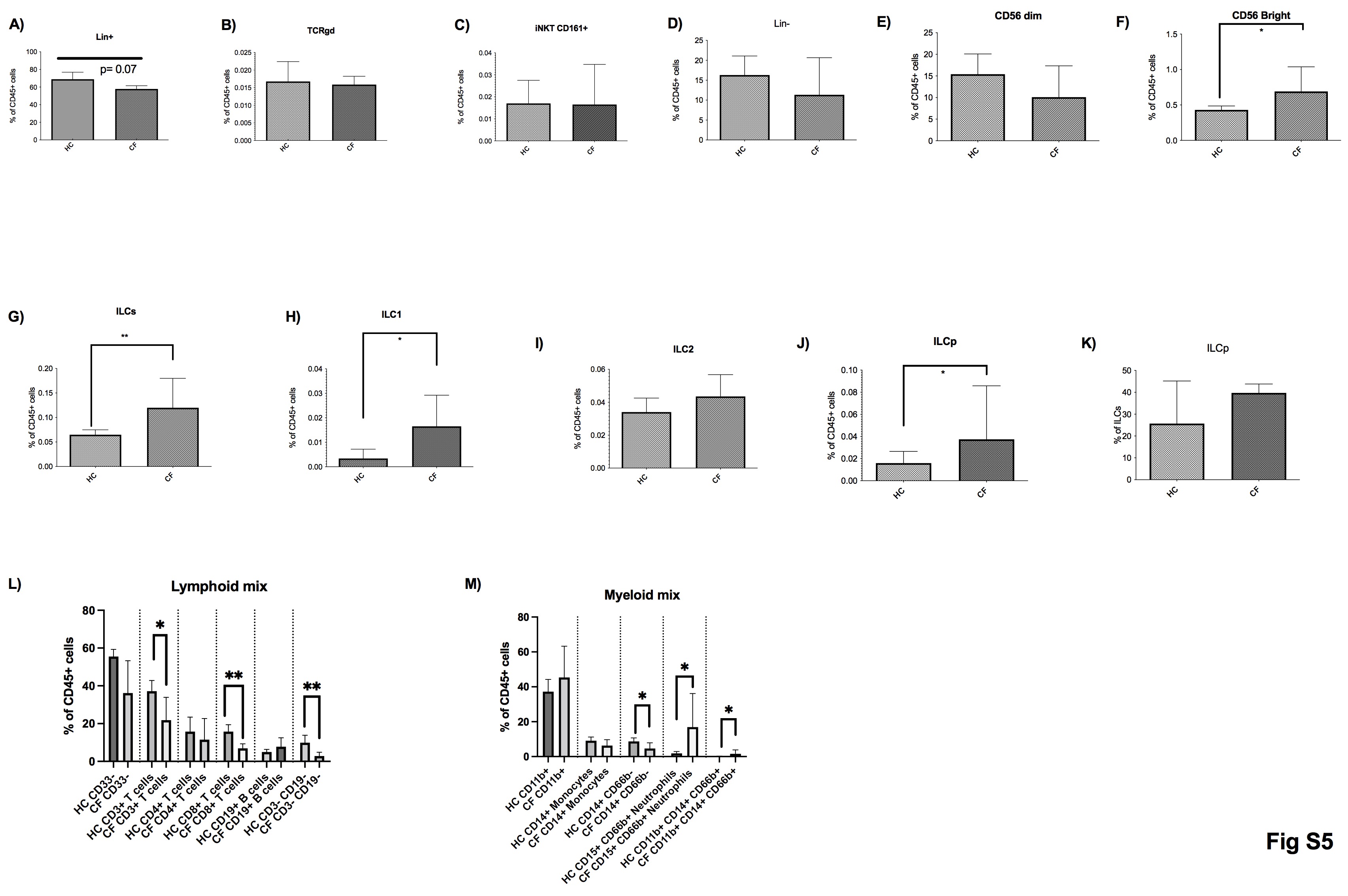

Supplement: Supplementary Figure S5 — FACS analysis of PBMCs markers in healthy controls (HC) and people with CF .The same analysis as in Figure 3 was performed, except that numbers are expressed as % relative to CD45+ cells. [file Image_5.jpeg]

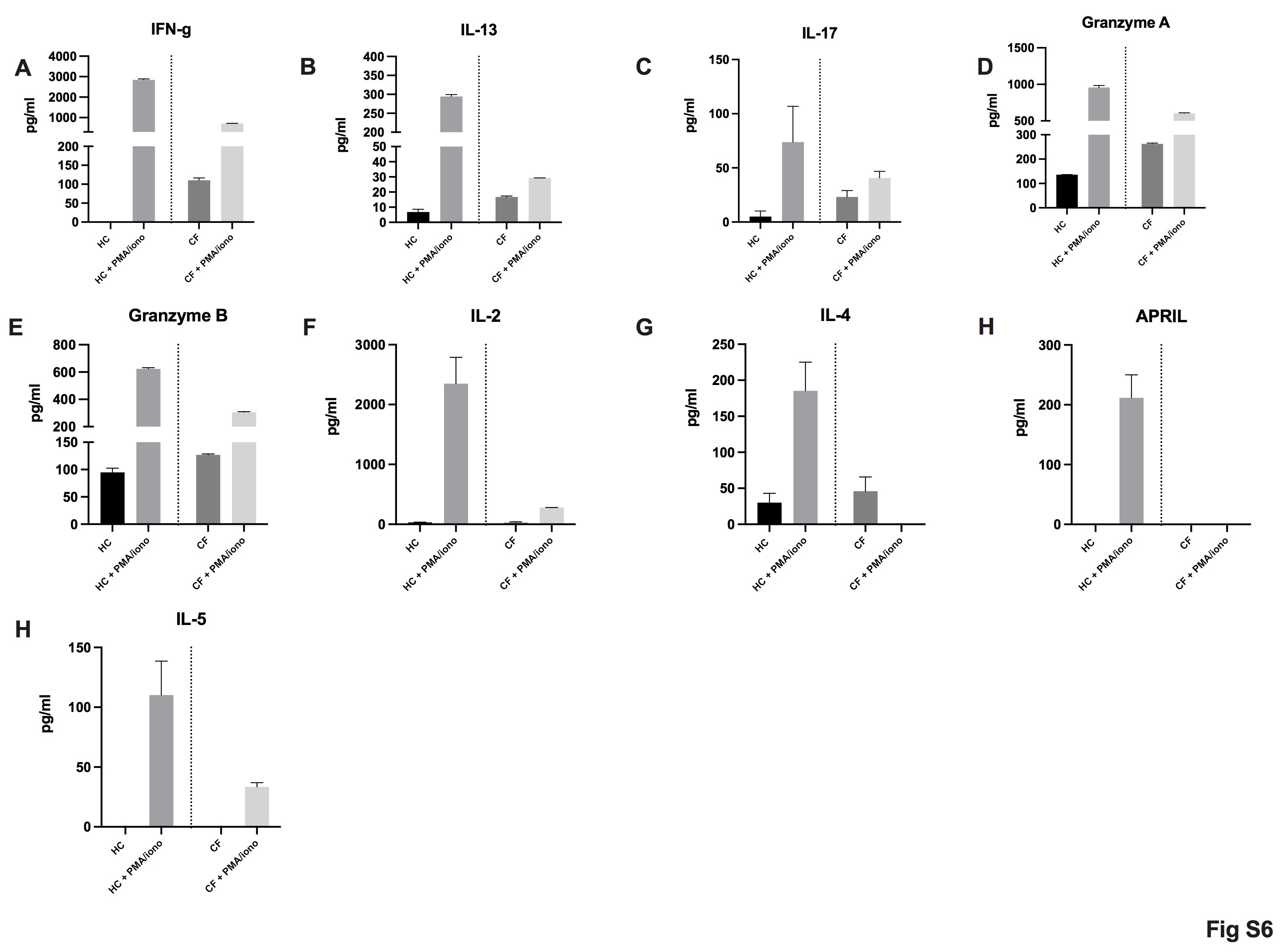

Supplement: Supplementary Figure S6 — Lymphoid cytokine production by PBMCs post PMA/ionomycin stimulation. HC (n= 3) and CF (n= 3) PBMCs were stimulated with PMA/iono during 4hrs as described in Figure 6 and in Materials and Methods. Supernatants were recovered and ‘lymphoid cytokines’ levels were analysed by Luminex [file Image_6.jpeg]

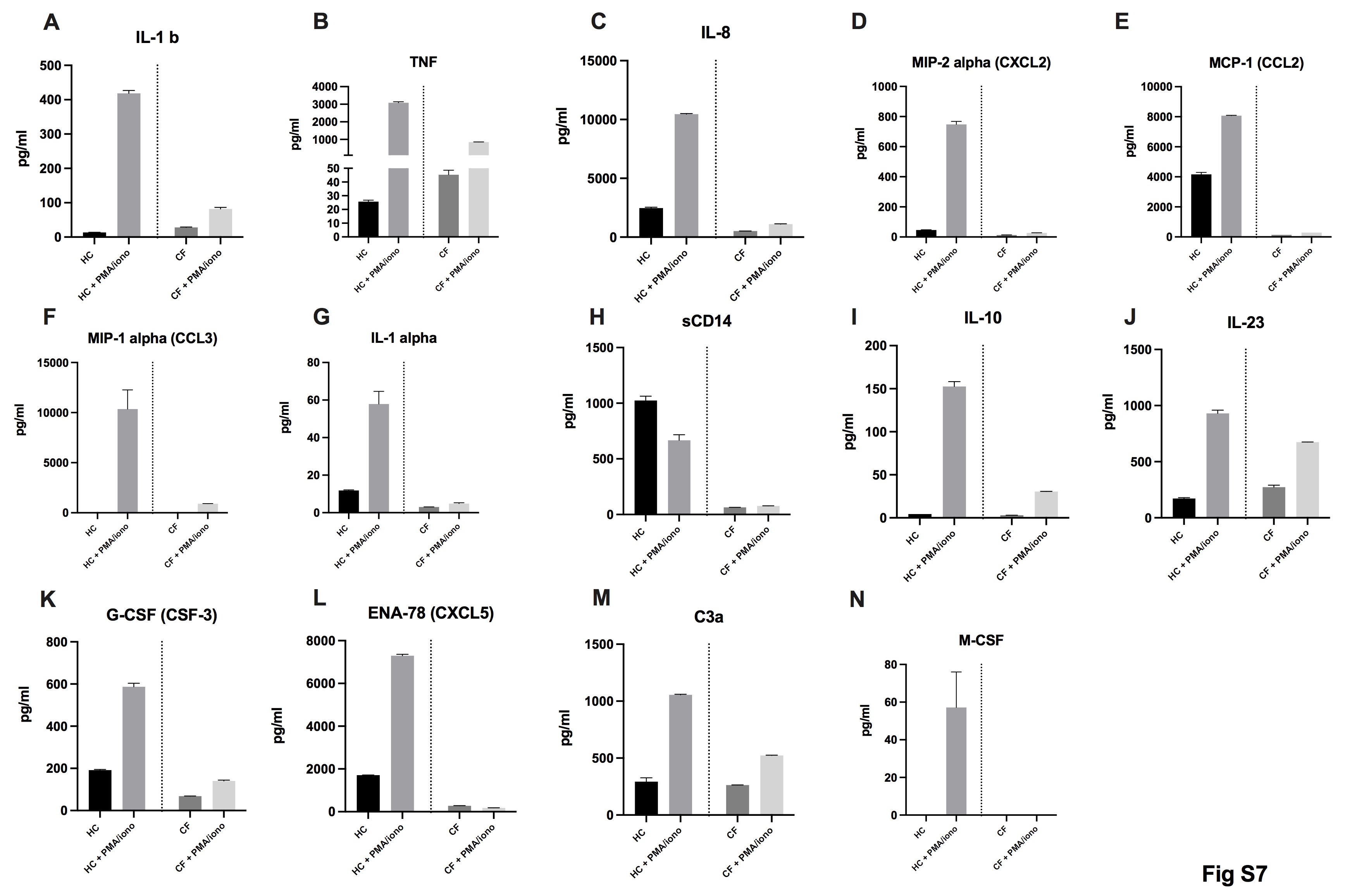

Supplement: Supplementary Figure S7 — Myeloid cytokine production by PBMCs post PMA/ionomycin stimulation. The same supernatants generated as explained in Supplementary Figure S6 were analysed by Luminex for assessing ‘myeloid cytokines’ levels. [file Image_7.jpeg]

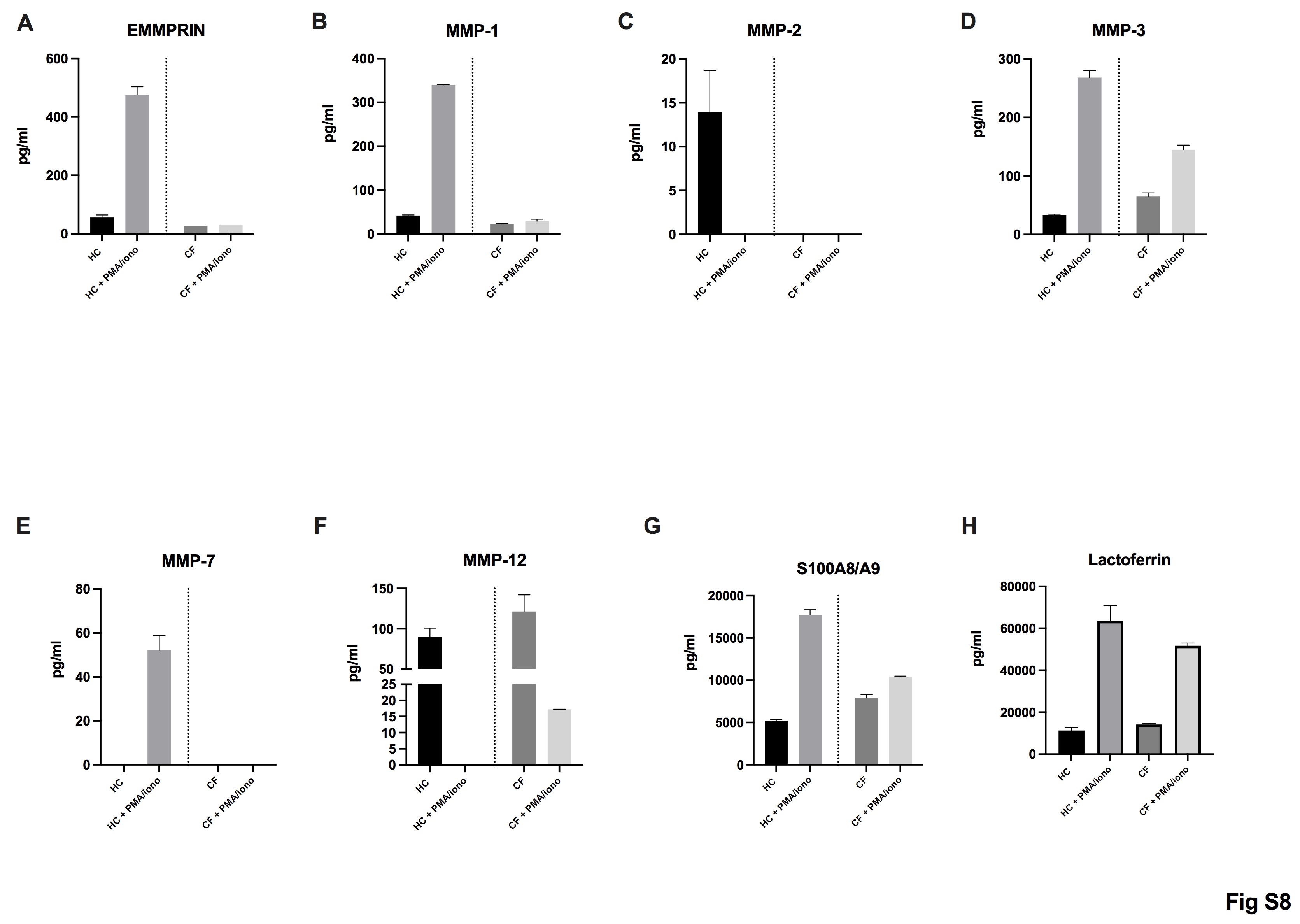

Supplement: Supplementary Figure S8 — Neutrophilic secretory granules markers production by PBMCs post PMA/ionomycin stimulation. The same supernatants generated as explained in Supplementary Figure S6 were analysed by Luminex for assessing ‘neutrophilic secretory granules’ levels. [file Image_8.jpeg]

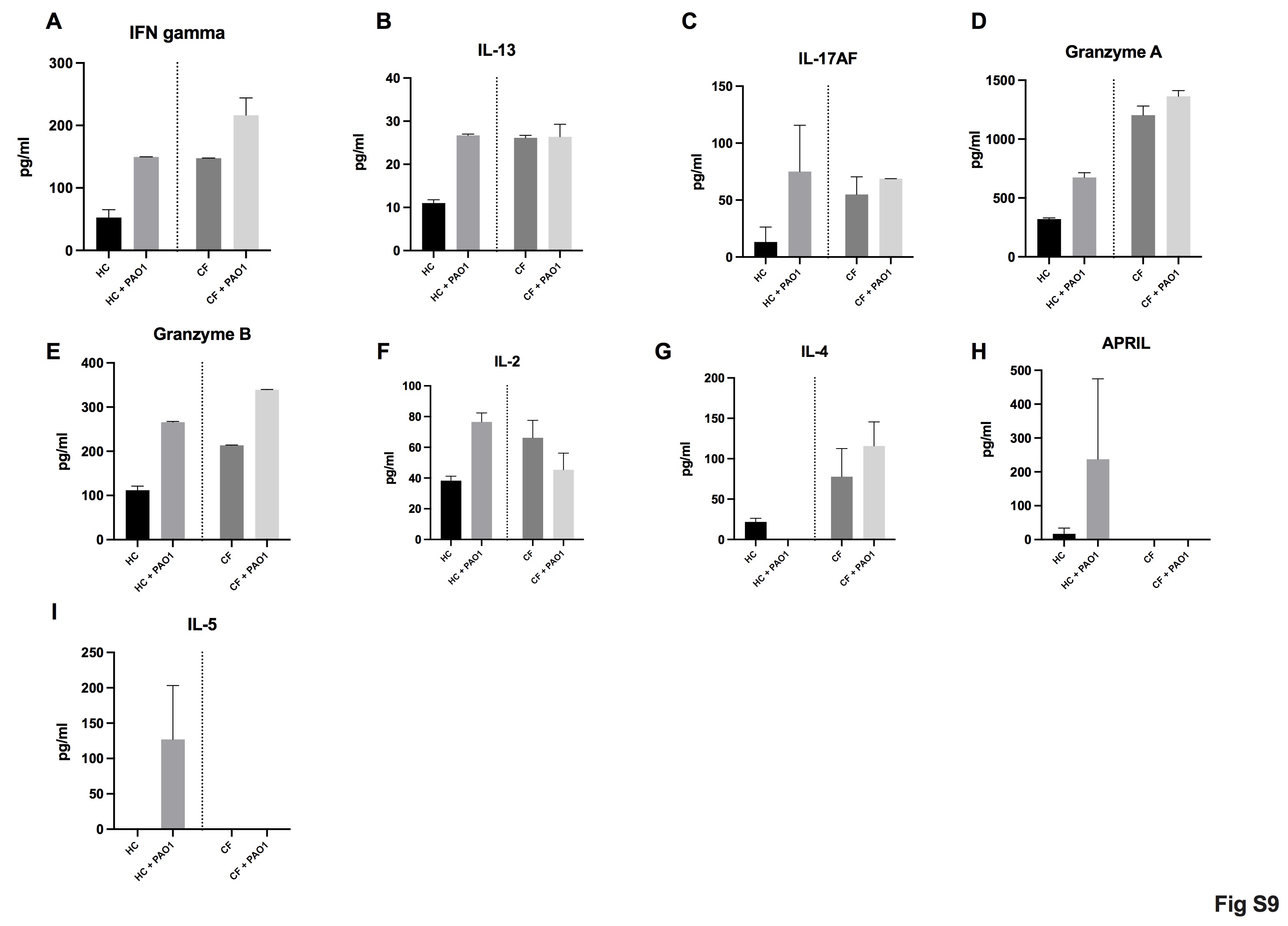

Supplement: Supplementary Figure S9 — Lymphoid cytokine production by PBMCs post live PAO1 infection. HC (n= 9) and CF (n= 22) PBMCs were infected with live PAO1 during 4hrs as described in Figure 7 and in Materials and Methods. Supernatants were recovered and ‘lymphoid cytokines’ levels were analysed by Luminex. [file Image_9.jpeg]

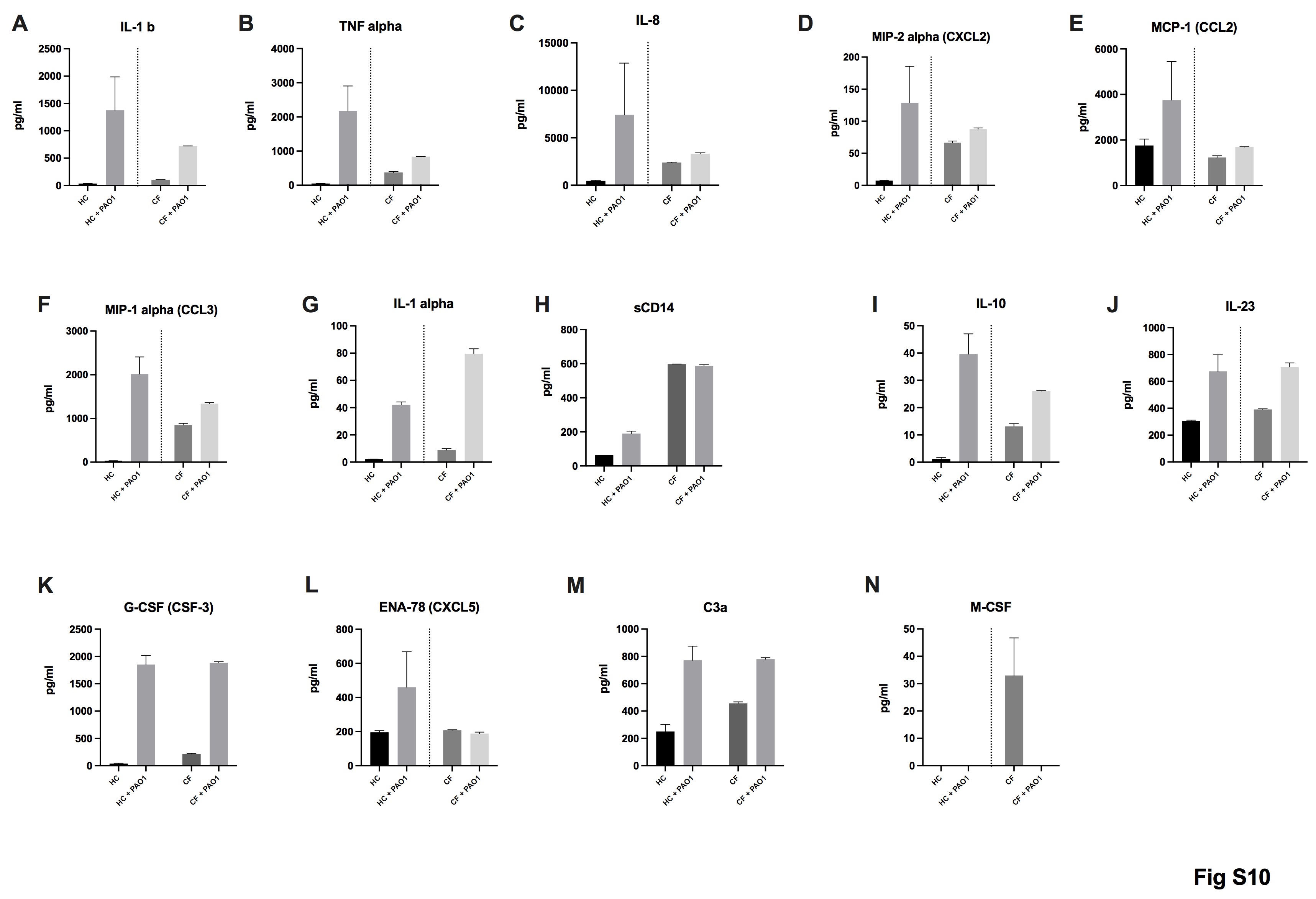

Supplement: Supplementary Figure S10 — Myeloid cytokine production by PBMCs post live PAO1 infection. The same supernatants generated as explained in Supplementary Figure S8 were analysed by Luminex for assessing ‘myeloid cytokines’ levels. [file Image_10.jpeg]

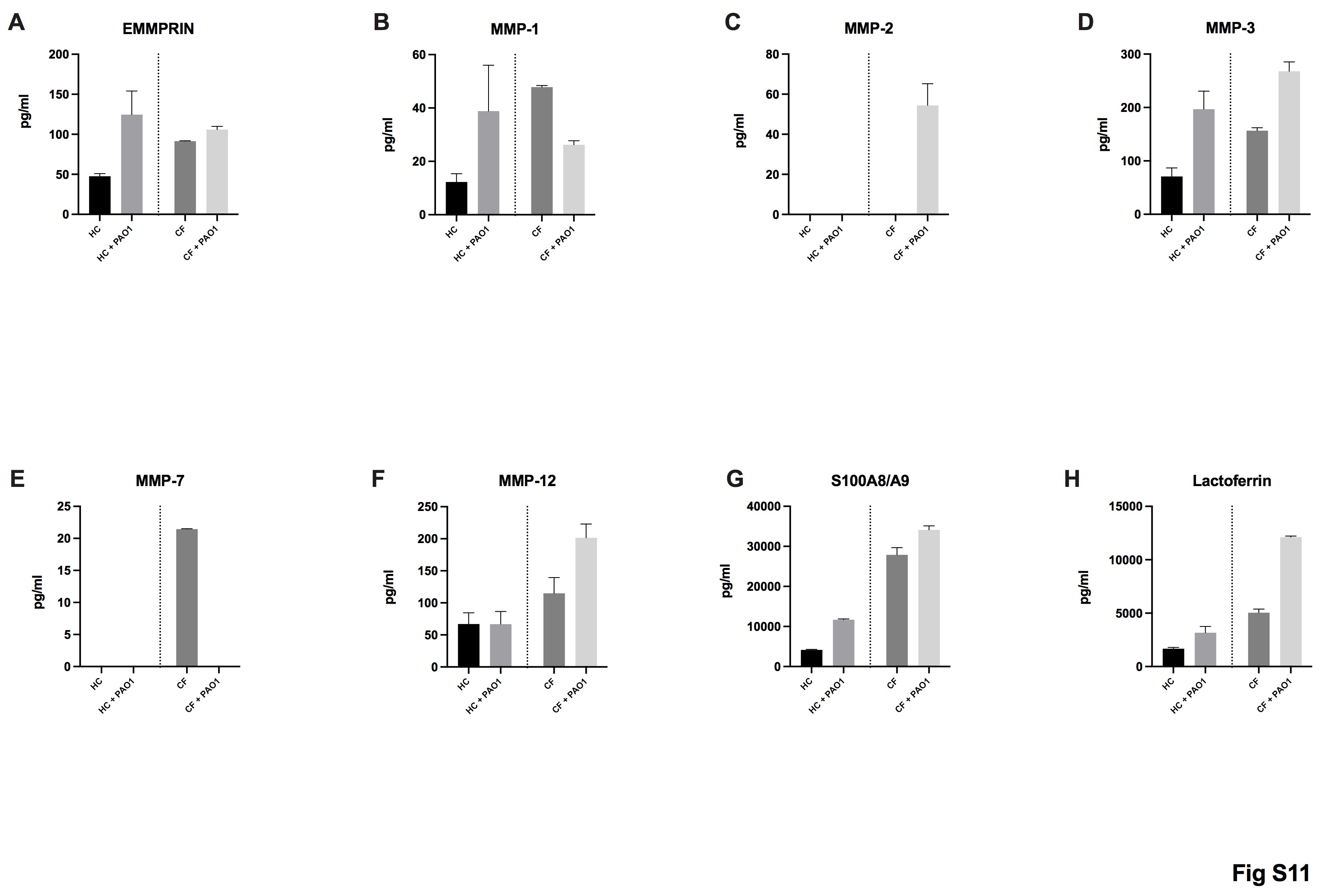

Supplement: Supplementary Figure S11 — Neutrophilic secretory granules markers production by PBMCs post live PAO1 infection. The same supernatants generated as explained in Supplementary Figure S9 were analysed by Luminex for assessing ‘neutrophilic secretory granules’ levels. [file Image_11.jpeg]
